# Effects of rTMS on swallowing function and neuroimaging features in post-stroke dysphagia

**DOI:** 10.3389/fnhum.2025.1573083

**Published:** 2025-12-03

**Authors:** Xuting Chen, Lianjie Ma, Mengdi Hou, Xudong Gu, Zhongli Wang, Yunhai Yao, Jianming Fu, Meihong Zhu, Jie Wang, Chaofan Wang, Xiaolin Sun, Ting Zhang, Xiaoqing Ma, Xinxin Song, Ming Zeng

**Affiliations:** 1 Department of Rehabilitation Medicine, The Second Affiliated Hospital of Jiaxing University, The Second Hospital of Jiaxing City, Jiaxing, Zhejiang, China; 2 Department of Radiology, Changshu Hospital Affiliated to Nantong University, Changshu, Jiangsu, China; 3 Jiaxing University Master Degree Cultivation Base, Zhejiang Chinese Medical University, Jiaxing, Zhejiang, China

**Keywords:** stroke, dysphagia, repetitive transcranial magnetic stimulation, resting-state fMRI, swallowing function

## Abstract

**Introduction:**

Dysphagia, or difficulty swallowing, is common after stroke and can lead to complications like malnutrition, aspiration pneumonia, and increased mortality. Recovery is driven by neural reorganization, yet traditional interventions focus on managing swallowing difficulties rather than restoring brain function. Neuromodulatory approaches like repetitive transcranial magnetic stimulation (rTMS) show potential for promoting brain plasticity and recovery. While rTMS has demonstrated efficacy in improving swallowing after stroke, few studies have explored its neural mechanisms at the brain level, as opposed to focusing on motor-evoked potentials recorded from peripheral muscles.

**Methods:**

This study examined the effects of 5 Hz rTMS on post-stroke dysphagia by targeting the contralesional mylohyoid cortical area. Resting-state fMRI was employed to investigate the neural correlates of rTMS effects. Local brain activity was measured using the amplitude of low-frequency fluctuation (ALFF), fractional amplitude of low-frequency fluctuation (fALFF), and percentage amplitude of fluctuation (PerAF), while network connectivity was assessed with graph theory analysis.

**Results:**

rTMS reduced spontaneous activity in the contralesional middle frontal gyrus and putamen, and in the ipsilesional insula and middle frontal gyrus (pars orbitalis), regions that were hyperactive at baseline in dysphagic patients. Altered network topology in the left medial superior frontal gyrus suggested connectivity reorganization.

**Conclusion:**

These preliminary findings support rTMS as a promising adjunct therapy for post-stroke dysphagia by inducing cortical plasticity, as demonstrated by changes in both regional activity and network topology. Further validation in studies with larger samples is needed.

## 1 Introduction

Dysphagia, a medical condition marked by difficulties in swallowing, frequently occurs during the acute phase of stroke and affects up to 80% of cases (Banda et al., 2022; Takizawa et al., 2016). Dysphagia can lead to a range of complications, including malnutrition, dehydration, reduced quality of life, prolonged hospital stays, increased risk of aspiration pneumonia, and higher mortality (Jones et al., 2020). Swallowing is a complex physiological process mediated by both the central and peripheral nervous systems (Sasegbon et al., 2024). At the brain level, swallowing is controlled by multiple cortical and subcortical structures, including the primary motor cortex, primary somatosensory cortex, insula, and others. These areas are functionally connected in separate groups within and between the two hemispheres, and damage to them may result in dysphagia (Cheng et al., 2022).

While the majority of patients may recover their swallowing ability within a few weeks after an acute stroke, dysphagia can impact long-term functional outcomes for those who continue to experience swallowing difficulties (Sreedharan et al., 2022). The natural recovery process of dysphagia is thought to be driven by neural reorganization that compensates for the brain damage caused by stroke (Sasegbon et al., 2024). Notably, neuroplastic compensatory changes have been observed in the contralesional half of the brain. For instance, Hamdy et al. (1998) observed an increased pharyngeal representation (as mapped by TMS) in the unaffected hemisphere of patients recovering from dysphagia after unilateral cortical stroke, whereas little change was noted in the affected hemisphere or in patients who were non-dysphagic or persistently dysphagic. Similarly, Fraser et al. (2002) found expanded pharyngeal representation and increased excitability in the unaffected hemisphere of acutely dysphagic stroke patients following electrical stimulation of the pharynx, which correlated with improvement in swallowing behavior.

Based on the above findings, treatment with neurostimulation techniques could be beneficial for post-stroke dysphagia, as they can promote the reorganization of the swallowing neural network in patients who would not naturally recover, while also facilitating recovery in those who compensate naturally (Cheng et al., 2022). Traditional interventions for neurogenic dysphagia—such as dietary modifications, behavioral rehabilitation exercises, and artificial feeding—were not designed to directly address neural damage and restore normal brain function (Georgiou et al., 2024; Sasegbon et al., 2024). Consequently, neuromodulatory techniques have been explored as additional therapeutic options. These techniques generally follow two approaches: peripheral nervous system stimulation, and direct stimulation of the brain using non-invasive methods such as repetitive transcranial magnetic stimulation (rTMS) and transcranial direct current stimulation (tDCS) (Georgiou et al., 2024).

Transcranial magnetic stimulation (TMS) utilizes electromagnetic induction properties to induce electric currents in the stimulated brain tissue (Barker et al., 1985). If the imposed electric field is strong enough to depolarize the membrane potential of a neuron above the firing threshold, an action potential will be fired. With rTMS, stimulation is delivered in trains of several TMS pulses. These pulses can be applied at low frequencies, such as 1 Hz, to induce neuronal suppression (with 1 Hz rTMS typically having inhibitory effects) or at higher frequencies (5–25 Hz) to increase neuronal excitability (Hoogendam et al., 2010). It is thought that rTMS modulates brain activity by inducing changes in synaptic plasticity through mechanisms like long-term depression (LTD) and long-term potentiation (LTP) (Hoogendam et al., 2010). LTP, an increase in the synaptic strength that could last for days or longer, could be induced by brief high-frequency stimulation. LTD, on the other hand, refers to a long-lasting weakening of neuronal synapses (Hoogendam et al., 2010). Randomized controlled trials (RCTs) have explored the efficacy of rTMS for treating post-stroke dysphagia in the acute, subacute, and chronic stages. A recent umbrella review of systematic reviews and meta-analyses, which included these RCTs, concluded that rTMS is likely to improve swallowing ability (Georgiou et al., 2024). However, the number of primary RCTs remains relatively small, despite the growing number of systematic reviews in the field (Georgiou et al., 2024).

The current study aimed to investigate the effectiveness of rTMS on post-stroke dysphagia in subacute patients, using resting-state fMRI (rs-fMRI) and clinical rating scales to assess the impact of the intervention. Rs-fMRI refers to the acquisition of fMRI data while the brain is “at rest,” meaning no tasks or external stimuli are involved. Low-frequency fluctuations (around 0.01 to 0.1 Hz) in the resting-state BOLD signal are temporally correlated between functionally related brain regions (e.g., motor cortices), suggesting that these spontaneous fluctuations are neurophysiologically meaningful (Biswal et al., 1995). Subsequent research suggested that low-frequency BOLD fluctuations may reflect integrative postsynaptic potentials in gray matter, supporting the notion that resting-state BOLD signals capture neuronal activity (Shmuel and Leopold, 2008). Therefore, by representing ongoing neuronal activity and functional connectivity, these fluctuations can provide insight into intrinsic brain functions and their alterations in neurological and psychiatric disorders (Fox and Raichle, 2007).

Several studies have employed rs-fMRI to explore the neural mechanisms of post-stroke dysphagia, revealing altered regional brain activity (Li et al., 2022; Long et al., 2019; Zeng et al., 2023) and functional connectivity (Dai et al., 2022; Li et al., 2014a, 2014b) in dysphagic patients compared to healthy controls and non-dysphagic stroke patients. However, few studies have evaluated the effect of rTMS on post-stroke dysphagia using rs-fMRI. One such study (Jiao et al., 2020) assessed patients with an average stroke onset of over 3 weeks, showing that rTMS applied over bilateral motor and frontal areas, in conjunction with conventional rehabilitation, led to changes in the amplitude of low-frequency fluctuation (ALFF) in the right basal ganglia and superior frontal gyrus when compared to sham rTMS.

In the present study, we stimulated the contralesional cortex of patients with high-frequency rTMS and examined its influence on the brain at both regional and network levels. The intensity of spontaneous low-frequency fluctuations was evaluated using a combination of metrics that quantify their amplitude, including ALFF, fractional ALFF (fALFF), and percentage amplitude of fluctuation (PerAF). These metrics aim to capture the strength of neuronal activity reflected in low-frequency BOLD signals and are complementary to one another. ALFF is calculated as the square root of the power spectrum within the low-frequency range (typically 0.01–0.08 Hz), but it is susceptible to physiological noise (Yu-Feng et al., 2007). fALFF, defined as the ratio of power in the low-frequency range (0.01–0.08 Hz) to that of the entire frequency range (0–0.25 Hz), normalizes ALFF to suppress physiological noise in cistern areas and enhance signals from cortical regions (Zou et al., 2008). PerAF, which measures the percent signal change per volume relative to the mean time series signal intensity, has demonstrated better short- and long-term test–retest reliability than both ALFF and fALFF (Jia et al., 2020). Brain regions with abnormal ALFF, fALFF, or PerAF were then selected as regions of interest (ROIs) for subsequent graph theory analysis. By examining the system’s topological properties—that is, the arrangement of functional connections among brain regions—graph theory offers a better understanding of brain network organization than functional connectivity analysis alone (Minati et al., 2013). We expected that rTMS would modulate both local activity and network topology. Our findings may contribute to the evidence base for evaluating the therapeutic effects of rTMS in post-stroke dysphagia and shed light on its potential neural correlates.

## 2 Methods

### 2.1 Participants

This study was conducted at the Rehabilitation Medicine Centre of Jiaxing Second Hospital. Between January 2022 and October 2024, 47 patients hospitalized for their first stroke with dysphagia were recruited (voluntary participation) and randomly assigned to either the rTMS treatment group (22 patients) or the sham rTMS group (25 patients) using a random number table. Healthy controls were individuals who voluntarily underwent MRI scans.

Inclusion criteria for patients were as follows: (1) Diagnosis of stroke meeting the revised criteria set by the 4th National Stroke Conference (Liu et al., 2023), and confirmed by imaging; (2) First-time onset, age ≤ 80 years, with unilateral cerebral hemisphere or brainstem stroke; (3) Conscious upon admission, with stable vital signs; (4) Exhibiting signs of dysphagia, such as coughing while drinking water or having difficulty swallowing liquids or food, along with an EAT-10 score ≥ 3, an SSA score ≥ 24, and an MMASA score ≤ 95; (5) Disease duration ≤ 6 months, able to cooperate with examinations and treatment; (6) Informed consent obtained from both the patient and their family prior to study inclusion; (7) Right-handed with normal vision.

Exclusion criteria for patients were: (1) Dysphagia caused by other neurological conditions, such as traumatic brain injury, head and neck tumors, psychiatric disorders, Parkinson’s disease, or motor neuron diseases; (2) Lesions in both cerebral hemispheres or both sides of the brainstem, or primary lesion not confined to one side; (3) Damage to vital organs or severe underlying conditions, such as malignant tumors; (4) Tendency toward coma or bleeding; (5) Contraindications for rTMS (intracranial metal implants, pacemaker implants, history of epilepsy, pregnancy, breastfeeding, or intracranial drainage tubes); (6) Onset of a major illness or life-threatening condition during treatment; (7) Excessive head movement during MRI scanning that interferes with data collection.

We included 18 healthy volunteers who were aged between 20 and 80 years, right-handed, had normal vision, and had no history of neurological or psychiatric disorders, brain injury, or contraindications for MRI.

The study was approved by the Ethics Committee of the Second Affiliated Hospital of Jiaxing University (JXEY-2022SW014, JXEY-2023JX047) and conducted in accordance with the Declaration of Helsinki. It was prospectively registered (ChiCTR2100044993) and retrospectively registered (ChiCTR2500108286) with the Chinese Clinical Trial Registry. We obtained two ethics approval numbers (and correspondingly two trial registration numbers) because when the initial approval expired, we had not yet recruited enough patients. Therefore, we applied for and received a second approval to continue recruitment.

### 2.2 Clinical assessments

Swallowing function was assessed in all patients before and after treatment using the following scales: the Eating Assessment Tool (EAT-10) (Belafsky et al., 2008), the Standardized Swallowing Function Assessment Scale (SSA) (Perry, 2001), and the Modified Mann Assessment of Swallowing Ability (MMASA) (Antonios et al., 2010). Pre- and post-treatment assessments of swallowing function were conducted on the same days as the MRI scans. In addition, functional independence, which reflects stroke severity, was assessed at baseline using the Barthel Index for Activities of Daily Living (BI-ADL, 10 items) (Mahoney and Barthel, 1965).

The EAT-10 consists of 10 questions related to swallowing difficulties, with each question rated on a scale from 0 to 4, where 0 indicates normal swallowing and 4 indicates severe dysfunction. The total possible score is 40, with a score of 3 or higher suggesting swallowing dysfunction. The SSA is specifically designed to assess swallowing function and has three components: (1) a clinical examination assessing consciousness, head and trunk control, respiration, lip closure, soft palate movement, laryngeal function, pharyngeal reflex, and spontaneous coughing, with a total score ranging from 8 to 23; (2) a swallowing test where the patient swallows 5 mL of water three times while observing laryngeal movement, repetitive swallowing, stridor, and post-swallowing laryngeal function, with a score between 5 and 11 points; (3) an additional swallowing test with 60 mL of water, assessing swallowing time and coughing, yielding a score between 5 and 12 points. The total SSA score ranges from 18 to 46, with lower scores indicating better swallowing function. The MMASA includes 12 items evaluating consciousness, cooperation, respiration, expressive language disorders, listening comprehension, speech articulation disorders, saliva control, tongue muscle range and strength, pharyngeal reflex, cough reflex, and soft palate movement. The total score ranges from 19 to 100, with lower scores indicating poorer swallowing ability.

### 2.3 rTMS protocol

We used the RT-50 Navigation Transcranial Magnetic stimulator (Sichuan Junjian Wanfeng Medical Equipment Co., Ltd) with a maximum magnetic field strength of 2.5 T and an ‘8’-shaped coil with a 10 mm inner diameter and a 50 mm outer diameter. First, the patient’s resting motor threshold (RMT) was determined. The patient was instructed to relax and lie flat on the treatment bed. The skin was cleaned with alcohol to remove oils, enhancing conductivity between the surface electrodes and the skin. Two surface electrodes were then placed on the patient’s mylohyoid muscles. The 10–20 electrode placement system was used to locate the Cz point on the head, defined as the intersection of the line connecting the nasion and inion with that connecting the two ears. The coil was positioned 2–4 cm anterior and 4–6 cm lateral to the vertex on the contralesional cerebral hemisphere. The coil was gently moved within this region while maintaining tangency to the scalp to identify the location that elicited the largest motor evoked potential (MEP) from the mylohyoid muscle. This position was considered the optimal stimulation site. After locating the site, the intensity was gradually reduced. RMT was defined as the minimum stimulation intensity, expressed as a percentage of maximum stimulator output, that elicited at least three of five consecutive MEPs ≥ 50 μV.

For rTMS treatment, the coil was placed over the contralesional cerebral hemisphere at the identified stimulation site for the mylohyoid muscle. The coil was kept tangential to the skull surface. Stimulation was delivered at 80% of RMT with a frequency of 5 Hz. Each 2-s stimulation was followed by a 10-s interval. The rTMS session lasted 20 min, delivering a total of 1,000 pulses. Treatment was given once daily, 6 days per week, with a 1-day break, for 2 consecutive weeks.

Sham rTMS was delivered using the same stimulation site and parameters as rTMS. The protocol was identical to that of the rTMS group. However, during treatment, the coil was positioned perpendicularly to the skull at the stimulation site, thus not delivering any actual stimulation.

Patients began rTMS treatment within 1 week (range: 1–6 days) of their initial MRI scan. Due to practical constraints, post-treatment MRI scans were not always performed immediately after completing the treatment protocol, with delays ranging from 1 to 20 days. Time interval data were unavailable for patients in the sham rTMS group. All patients were blinded to group assignments, adhering to a single-blind design.

### 2.4 Traditional swallowing therapy

This included indirect training, such as active or passive functional training of the mouth, face, and tongue muscles, various sensory stimulation, oral exercises, and assistive techniques; and direct feeding training, such as guidance on the eating environment, positioning for swallowing, selection of foods with different textures and flavors, the optimal bite size for swallowing, and the removal of pharyngeal residue. Each treatment session lasted 30 min, once per day, 6 days a week. Traditional therapy was administered following active or sham rTMS treatment. All patients received traditional swallowing therapy and pharmacological treatment.

### 2.5 rs-fMRI image acquisition

Both patient groups underwent resting-state fMRI scans before and after intervention. Scanning was conducted using a Philips 3.0T superconducting MRI system (Netherlands), with a standard 32-channel phased-array head coil. During scanning, participants were instructed to close their eyes, relax, and remain still. Foam pads were used to stabilize the head, and noise-canceling headphones were provided to minimize auditory distractions.

The scanning protocol consisted of two steps: resting-state functional MRI and structural MRI. Resting-state fMRI was acquired using an echo-planar imaging (EPI) sequence with 46 axial slices (2.5 mm thick, 0.5 mm gap); repetition time (TR) = 2000 ms, echo time (TE) = 20 ms, flip angle = 90°, field of view (FOV) = 240 mm × 240 mm, and acquisition matrix = 96 × 96.

High-resolution T1-weighted images were acquired using a fast gradient echo sequence in the sagittal plane, covering the entire brain with 170 slices, each 1 mm thick, without slice gaps; TR = 7.9 ms, TE = 3.5 ms, FOV = 256 mm × 256 mm, and acquisition matrix = 256 × 256, voxel size = 1 mm × 1 mm × 1 mm. The total scan duration was 5 min and 2 s.

### 2.6 Rs-fMRI data processing

#### 2.6.1 Preprocessing

Neuroimaging data were processed on a Windows 11 computer (Version 24H2) equipped with a 13^th^ Gen Intel® Core™ i5-13500 processor (2.50 GHz, 14 cores) and 16 GB of RAM. Resting-state fMRI data were preprocessed using the Resting-State fMRI Data Analysis Toolkit plus (RESTplus) V1.27^1^ (Jia et al., 2019) based on SPM12^2^ in MATLAB R2017b (The MathWorks Inc., Natick, MA, USA). Preprocessing steps included (1) converting DICOM images to NIFTI format; (2) discarding the first 10 volumes; (3) slice time correction (Yan and Zang, 2010); (4) realignment using rigid body transformation and exclusion of participants whose head motion exceeded 3 mm of translation or 3° of rotation (Yan and Zang, 2010; Power et al., 2015); (5) spatial normalization of the realigned functional images to Montreal Neurological Institute (MNI) space using the new segment method, and resampling to 3 mm isotropic voxels; (6) spatial smoothing with a Gaussian kernel of 6 mm full-width at half-maximum (Lv et al., 2019a, 2019b; Petersson et al., 1999); (7) linear detrending (Turner, 1997; Lowe and Russell, 1999); (8) regressing out nuisance signals, including (a) the Friston-24 head motion parameters to remove residual motion-related artifacts (Friston et al., 1996), and (b) cerebrospinal fluid and white matter signals (Fox et al., 2005). The “add mean back” option was also selected. Lastly, band-pass filtering (0.01–0.08 Hz) was applied prior to PerAF and functional connectivity analyses.

#### 2.6.2 Post-processing

##### 2.6.2.1 Regional brain activity

ALFF, fALFF and PerAF were computed in RESTplus V1.27. Voxel-wise preprocessed time series were band-pass filtered (0.01–0.08 Hz) and converted to the frequency domain via fast Fourier transform (FFT) to obtain the power spectrum. The square root of the power spectrum was computed at each frequency and then averaged across the 0.01–0.08 Hz band to generate the ALFF value for each voxel (Yu-Feng et al., 2007). Voxel-wise fALFF was calculated as the ratio of the sum of amplitudes in the 0.01–0.08 Hz band to that of the full frequency range (0–0.25 Hz) (Zou et al., 2008).

PerAF for each voxel is given by the following formula: where *Xi* is the signal intensity at the *i*^th^ volume, *μ* is the mean signal intensity of the time series, and *n* is the total number of volumes (Jia et al., 2020).


$$\text{PerAF}=\frac{1}{n}\sum_{i=1}^{n}|\frac{X_{i}-\mu }{\mu }|\times 100\%$$

$$\mu =\frac{1}{n}\sum_{i=1}^{n}X_{i}$$

For standardization, the ALFF, fALFF and PerAF values of each voxel were divided by the corresponding global mean value within a whole-brain mask.

Because all patients in this study had unilateral stroke lesions, the standardized ALFF, fALFF, and PerAF images of patients with right-sided lesions were flipped along the left–right axis, so that all lesions appeared on the left hemisphere. In total, the images of 6 patients (both pre- and post-treatment) were flipped prior to statistical analysis.

##### 2.6.2.2 Graph theory analysis

Graph theory analysis was conducted to assess the effects of active and sham rTMS on the functional organization of brain connectivity. For patients with right-sided brain lesions (n = 6), the preprocessed rs-fMRI images had been left–right flipped prior to constructing functional connectivity matrices.

###### 2.6.2.2.1 Functional connectivity matrix construction and network analysis

Using the MNI coordinates of the peak *t* values from the preceding analysis (i.e., 21 stroke patients vs. 14 healthy controls; see section 2.7.2 and Table 1), we extracted the mean time series of 18 ROIs (radius = 6 mm) from the preprocessed images. ROI-to-ROI functional connectivity analysis was then performed in RESTplus V1.27 to obtain the Pearson correlation coefficient matrix for each patient. This generated two 18 × 18 matrices per patient: one for the pre-intervention scan and one for the post-intervention scan. Thus, graph analysis was restricted to functionally abnormal regions identified in patients, enabling us to examine how rTMS modulated interactions specifically among disease-relevant areas. This targeted approach may better capture pathophysiologically meaningful changes than nonspecific, brain-wide alterations.

**Table 1:** Differences in ALFF, fALFF and PerAF between dysphagic stroke patients (all patients combined) at baseline and healthy controls (HC).

Outcome	Brain region (aal)	Cluster size	Peak *t* (*p*) value	MNI coordinate (mm)		
				x	y	z
ALFF	Fusiform_R	277	4.0969 (<0.0005)	39	−30	−15
	Occipital_Sup_R	1974	−5.5686 (<0.0001)	21	−93	21
	Putamen_L	272	3.9994 (<0.0005)	−21	18	6
	Putamen_R	260	4.9617 (<0.0001)	27	15	12
	Frontal_Mid_R	2,554	5.6802 (<0.0001)	42	27	33
	Supp_Motor_Area_R	451	−4.9161 (<0.0001)	3	3	78
fALFF	Cerebellum_8_R	154	5.5958 (<0.0001)	27	−60	−45
	Temporal_Inf_R	419	4.7777 (<0.0001)	54	−30	−18
	SupraMarginal_L	1,428	−5.741 (<0.0001)	−54	−21	15
	Cuneus_R	2,650	−7.0853 (<0.0001)	15	−96	9
	Postcentral_R	534	−4.9817 (<0.0001)	66	−9	18
	Insula_L	166	5.2001 (<0.0001)	−33	6	18
	Supp_Motor_Area_R	426	4.3612 (<0.0005)	15	18	63
	Postcentral_R	211	−4.9409 (<0.0001)	33	−30	51
PerAF	Occipital_Sup_R	2077	−5.6677 (<0.0001)	21	−90	21
	Rolandic_Oper_R	182	−3.3985 (<0.005)	66	6	12
	Frontal_Mid_Orb_L	236	3.9554 (<0.0005)	−42	57	−6
	Frontal_Sup_Medial_L	185	3.4829 (<0.005)	−6	39	39
	Frontal_Mid_R	793	4.9288 (<0.0001)	42	27	33

Comparisons were made by subtracting HC from the patient group. Fusiform_R = right fusiform; Occipital_Sup_R = right superior occipital gyrus; Putamen_L = left putamen; Putamen_R = right putamen; Frontal_Mid_R = right middle frontal gyrus; Supp_Motor_Area_R = right supplementary motor area; Cerebellum_8_R = lobule VIII of right cerebellar hemisphere; Temporal_Inf_R = right inferior temporal gyrus; SupraMarginal_L = left supramarginal gyrus; Cuneus_R = right cuneus; Postcentral_R = right postcentral gyrus; Insula_L = left insula; Rolandic_Oper_R = right rolandic operculum; Frontal_Mid_Orb_L = left middle frontal gyrus, pars orbitalis; Frontal_Sup_Medial_L = left medial superior frontal gyrus.

Network metrics were computed in GRETNA V2.0.0 (Wang et al., 2015). The functional connectivity matrices produced by RESTplus served as inputs for GRETNA. Hence, there were 18 nodes in the graph. The edges of the networks were the weighted correlation coefficients (i.e., the Pearson *r* values). We applied a range of sparsity thresholds to the correlation matrices to remove weak or spurious connections in the functional connectivity graphs (Adamovich et al., 2022). The sparsity of a network is defined as the ratio of the actual number of edges to the maximum possible number of edges. A commonly used propotional-density window is 0.05–0.5 (Liu Y. et al., 2022; Ning et al., 2022; Wei et al., 2024). The minimum threshold of 0.05 (i.e., retaining only the top 5% strongest connections between regions) yields a graph in which most regions remain interconnected, providing sufficient network connectivity for meaningful analysis (Finc et al., 2017). The upper bound of 50% connection density was chosen to preserve the small-world properties (*σ* > 1) of the functional brain network, because σ approaches 1 with increasing graph density, indicating reduced small-world structure and a shift toward random graph behavior (Hlinka et al., 2012; Wei et al., 2024). Accordingly, we employed a threshold range of 0.05 to 0.5, in 0.05 increments, to ensure adequate network connectivity while preserving small-world organization. The number of random networks was set to the default value of 100. Furthermore, only positive edges were included in the graph theory analysis due to the debate surrounding negative functional connectivity (Fox et al., 2009; Murphy et al., 2009).

###### 2.6.2.2.2 Network metrics

Global and nodal graph metrics were computed at each sparsity level to investigate potential changes in network topology after treatment. The global metrics included small-world propensity (Watts and Strogatz, 1998) and network efficiency parameters (global efficiency and local efficiency) (Latora and Marchiori, 2001). Briefly, a small-world network is defined as having clustering similar to a regular lattice and path length similar to a random network (Telesford et al., 2011). Efficiency is the reciprocal of the shortest path length, where the shortest path length measures the minimum number of edges connecting a pair of nodes. Global efficiency quantifies the average communication efficiency across the entire network, whereas local efficiency reflects the efficiency of communication among the immediate neighbors of each node. The local efficiency of the whole network is then calculated as the average of these efficiencies across all nodes’ neighborhoods.

Nodal metrics included clustering coefficient (sparsity of the sub-network comprising a node’s first neighbors), shortest path length (average minimum number of edges connecting a node to all other nodes), efficiency (average communication efficiency between a node and all other nodes), local efficiency (communication efficiency within the sub-network formed by a node’s immediate neighbors), degree centrality (number of direct connections a node has), and betweenness centrality (proportion of shortest paths in the network that pass through a node).

### 2.7 Statistical analysis

#### 2.7.1 Demographic and clinical data

Statistical analysis of demographic and clinical data was performed using GraphPad Prism 9 (GraphPad Software, Boston, MA, USA^3^), except for the chi-square test, which was conducted in SPSS Version 26.0 (SPSS, Inc., Chicago, IL, USA). Statistical significance was set to *p* < 0.05.

#### 2.7.2 Regional brain activity

##### 2.7.2.1 Comparing dysphagic stroke patients with healthy controls

First, the pre-treatment (baseline) data of all dysphagic stroke patients (11 from the rTMS group + 10 from the sham rTMS group) were combined and compared with healthy controls to identify brain regions showing significant differences in ALFF, fALFF, or PerAF. Independent-samples *t*-tests were performed in RESTplus V1.27. Baseline age, sex, and years of education were used as covariates in the statistical comparison between patients and healthy controls. The Gaussian Random Field (GRF) theory was applied to correct for multiple comparisons using voxel-level *p* < 0.05 and cluster-level *p* < 0.05 (Xie et al., 2023; two-tailed, vertex-connected). A gray matter mask based on the 116-region Automated Anatomical Labeling (AAL) atlas was used for statistical comparison and multiple comparisons correction. As a supplementary analysis, mean framewise displacement (FD; Jenkinson et al., 2002) was also included as a covariate to examine whether additional control of head motion substantially influenced the results.

##### 2.7.2.2 Comparing pre- and post-treatment data

Next, we extracted standardized ALFF, fALFF and PerAF values from brain areas that showed significant pre-treatment differences between patients and controls (Table 1), using the MNI coordinates of the peak *t-*value as the center and a 6 mm radius. The paired *t*-test (or Wilcoxon matched-pairs signed rank test) was performed in GraphPad Prism 9 to compare pre- and post-treatment ALFF, fALFF, and PerAF within each patient group in these significant regions. A Bonferroni correction was then applied (*α* = 0.0026). This analysis examines whether active and sham rTMS modulated brain regions showing abnormal activity at baseline in patients (Langenecker et al., 2007; Ritchey et al., 2011).

#### 2.7.3 Graph theory analysis

Statistical comparisons of graph metrics were performed in GRETNA V2.0.0. Paired *t*-tests assessed the effects of rTMS and sham rTMS on network metrics within each patient group. Results were Bonferroni-corrected (*α* = 0.00043). For all network metrics, the area under the curve (AUC) was used for statistical comparisons. The AUC was calculated across all sparsity thresholds for each node.

The reproducibility of our findings was validated by reanalyzing all graph-theoretical metrics using a different proportional density window and finer threshold steps (He et al., 2021). Specifically, we adopted a threshold range from 0.05 to 0.4 in 0.02 increments (Wang et al., 2022). All results were Bonferroni-corrected (*α* = 0.00043).

#### 2.7.4 Correlational analysis

In the rTMS group, changes in rs-fMRI data (post- minus pre-intervention) in brain areas significantly affected by rTMS were correlated with changes in clinical scale ratings (EAT-10, SSA, and MMASA). Following Shapiro–Wilk tests for normality, Pearson correlations were used to assess linear relationships between rs-fMRI data and clinical ratings. All analyses were performed in GraphPad Prism 9, and Bonferroni correction (*α* = 0.0024) was applied.

#### 2.7.5 Linear regression

To examine whether the delay (1–20 days) between the completion of rTMS treatment and post-treatment assessments may have confounded the observed effects, we conducted linear regression analyses (after checking assumptions) with delay time as the predictor variable. The dependent variables were the significant post- minus pre-intervention changes, including changes in clinical scores (EAT-10, SSA, and MMASA), as well as reductions in ALFF (right middle frontal gyrus and putamen), fALFF (left insula), PerAF (right middle frontal gyrus and left middle frontal gyrus, pars orbitalis), and in clustering coefficient and local efficiency (left medial superior frontal gyrus). A Bonferroni correction was applied (*α* = 0.005) to adjust for multiple comparisons.

## 3 Results

### 3.1 Demographic and clinical data

**Figure 1:**
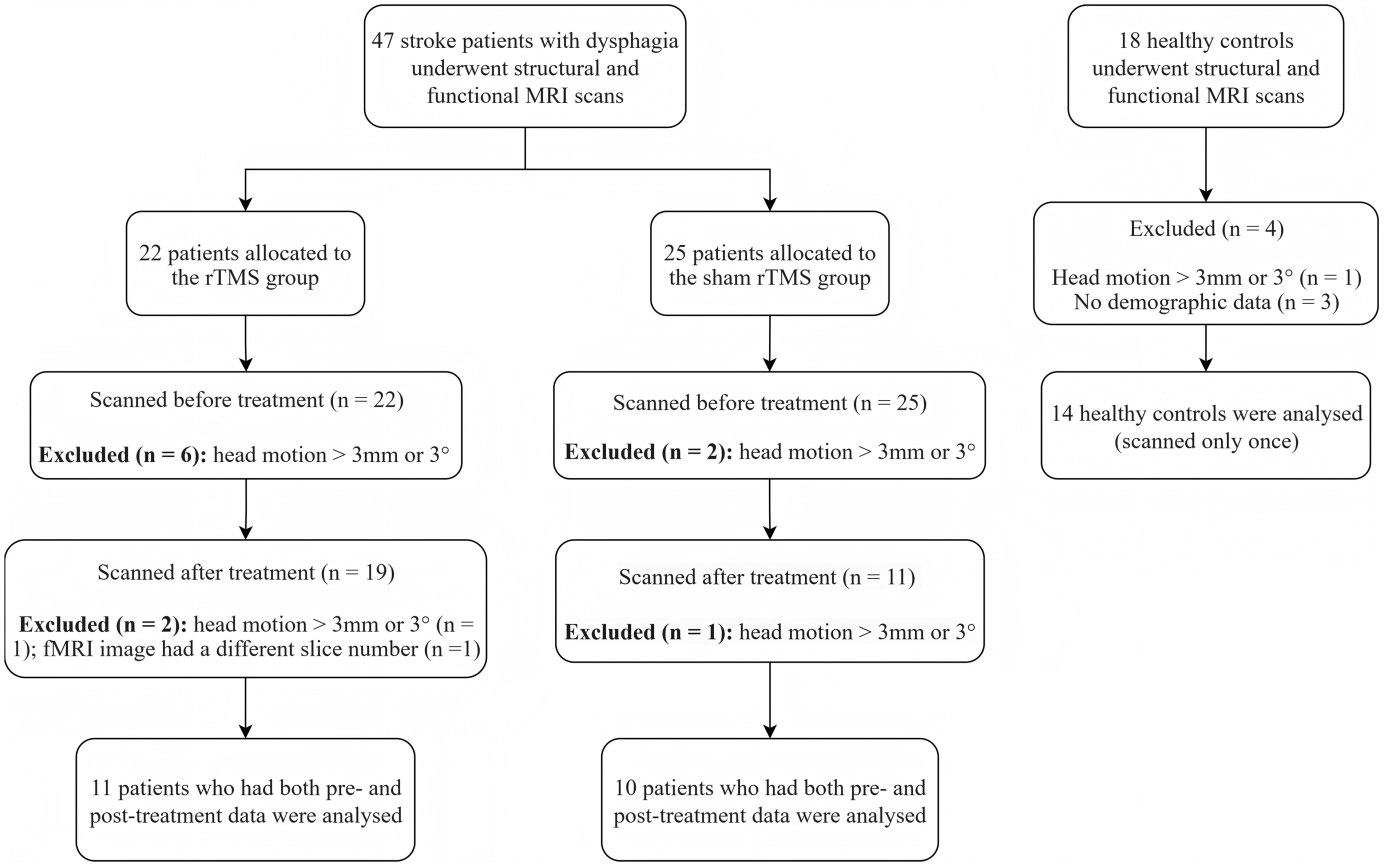
Flowchart of the participant inclusion and exclusion process.

Information on participant exclusion and inclusion is displayed in Figure 1. Forty-six patients with post-stroke dysphagia and 18 healthy volunteers underwent structural and functional MRI scanning. Of the patients, 22 and 25 were allocated to receive active and sham treatment, respectively. Eleven patients in the rTMS treatment group were excluded from analysis due to head motion, no post-treatment data or poor fMRI data quality. Fifteen patients in the sham rTMS treatment group were excluded because of head motion or no post-treatment data. Four healthy volunteers with large head movement or no demographic information were excluded. Consequently, the analysis included 11 patients in the active treatment group and 10 in the sham group—all of whom had both pre- and post-treatment images of sufficient quality—and 14 healthy controls. Notably, the sham rTMS group had a higher dropout rate than the active rTMS group. This may be because, despite the study’s single-blind design (i.e., patients were blinded to group allocation), some patients in the sham group may have realized during treatment that they were not receiving real stimulation and thus chose to withdraw.

Table 2 shows the demographic and clinical characteristics of the dysphagic stroke patients and healthy controls. Patients treated with rTMS and healthy controls differed significantly in age and years of education, and rTMS-treated patients were also significantly older than their sham-treated counterparts. As for comparisons within patient groups (i.e., pre- vs. post-treatment data), both groups had significantly decreased EAT-10 and SSA scores and increased MMASA scores at post-treatment. Comparisons between the active and sham rTMS groups showed no significant differences in EAT-10, SSA, or MMASA scores at baseline. However, after treatment, dysphagia ratings differed significantly between the two groups, with rTMS patients showing greater improvement. Further, there were no significant between-group differences in BI-ADL scores at baseline or in illness duration (days) at baseline, post-treatment, or in the pre–post change.

**Table 2:** Demographic and clinical characteristics of dysphagic stroke patients and healthy controls (HC).

Characteristics	Patients treated with rTMS (*n* = 11)		Patients treated with sham rTMS (*n* = 10)		Healthy controls (*n* = 14)	*p-*values for statistical tests	
	Baseline	Post-treatment	Baseline	Post-treatment		Between-subjects comparison	Within-subjects comparison
Demographics							
Sex (female, %)	5 (45.45%)		2 (20%)		8 (57.14%)	^**1**^*p* = 0.189	
Age (years, mean ± SD)	71.82 ± 8.29		58.10 ± 15.39		49.14 ± 13.04	^**2**^rTMS vs. HC: *p* < 0.001 Sham vs. HC: *p* = 0.211 rTMS vs. sham: *p* = 0.045	
Education (years, mean ± SD)	6.27 ± 1.42		8 ± 4.57		13.07 ± 4.60	^*3^rTMS vs. HC: *p* < 0.001 Sham vs. HC: *p* = 0.102 rTMS vs. sham: *p* = 0.399	
Clinical characteristics							
Days since stroke onset (mean ± SD)	35.36 ± 18.06	65.18 ± 25.41	37 ± 18.64	79.10 ± 32.17		^**4**^Pre-treatment: *p* = 0.840 ^**5**^Post-treatment: *p* = 0.229	
Affected hemisphere (left, %)	9 (81.82%)		6 (60%)			^**1**^*p* = 0.269	
Lesion location (*n*, %)							
Frontal, temporal, parietal and occipital lobes	1 (9.09%)		1 (10%)				
Frontal, temporal and parietal lobes	0		1 (10%)				
Frontal and parietal lobes	1 (9.09%)		0				
Parietal and occipital lobes	0		1 (10%)				
Frontal lobe	1 (9.09%)		0				
Periventricular (lateral)	1 (9.09%)		1 (10%)				
Basal ganglia	5 (45.45%)		5 (50%)				
Brainstem	2 (18.18%)		1 (10%)				
EAT-10	32.64 ± 2.50	15.45 ± 2.34	33 ± 2.11	23.60 ± 1.27		^**5**^Pre-treatment: *p* = 0.619 ^**5**^Post-treatment: *p* < 0.001	^**6**^rTMS: *p* < 0.001 ^**6**^Sham rTMS: *p* < 0.001
SSA	35.18 ± 2.86	22.36 ± 1.69	33.90 ± 1.91	30.20 ± 1.03		^**4**^Pre-treatment: *p* = 0.247 ^**5**^Post-treatment: *p* < 0.001	^**6**^rTMS: *p* < 0.001 ^**6**^Sham rTMS: *p* < 0.001
MMASA	69.18 ± 4.92	88.91 ± 2.47	66 ± 6.34	72.10 ± 6.66		^**4**^Pre-treatment: *p* = 0.212 ^**7**^Post-treatment: *p* < 0.001	^**6**^rTMS: *p* < 0.001 ^**6**^Sham rTMS: *p* < 0.001
BI-ADL	30 ± 19.75		37.50 ± 17.04			^**4**^*p* = 0.365	

1. Chi-square test; 2. One-way ANOVA and Tukey’s multiple comparisons test; 3. Kruskal-Wallis test and Dunn’s multiple comparisons test; 4. Two-sample *t*-test; 5. Mann–Whitney test; 6. Paired *t*-test; 7. Two-sample *t*-test with Welch’s correction. EAT-10 = Eating Assessment Tool–10 items; SSA = Standardized Swallowing Assessment; MMASA = Modified Mann Assessment of Swallowing Ability; BI-ADL = Barthel Index for Activities of Daily Living (10 items). * Data were reciprocal-transformed prior to statistical testing.

### 3.2 Regional brain activity

**Figure 2:**
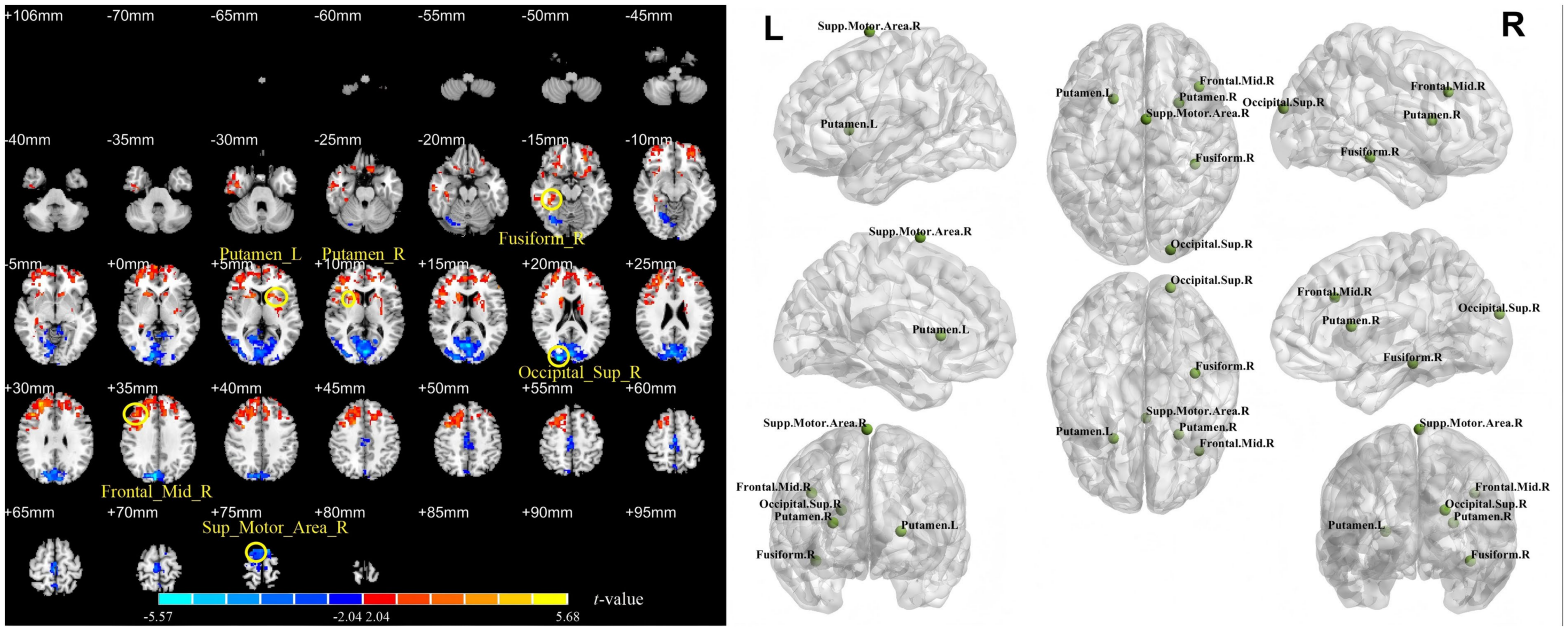
Differences in ALFF between stroke patients (11 in the rTMS group and 10 in the sham rTMS group) at baseline and healthy controls. Fusiform_R = right fusiform gyrus; Occipital_Sup_R = right superior occipital gyrus; Putamen_L = left putamen; Putamen_R = right putamen; Frontal_Mid_R = right middle frontal gyrus; Supp_motor_Area_R = right supplementary motor area. Each named area refers to the region containing the voxel with the peak *t*-value (i.e., the greatest difference) within its respective cluster.

**Figure 3:**
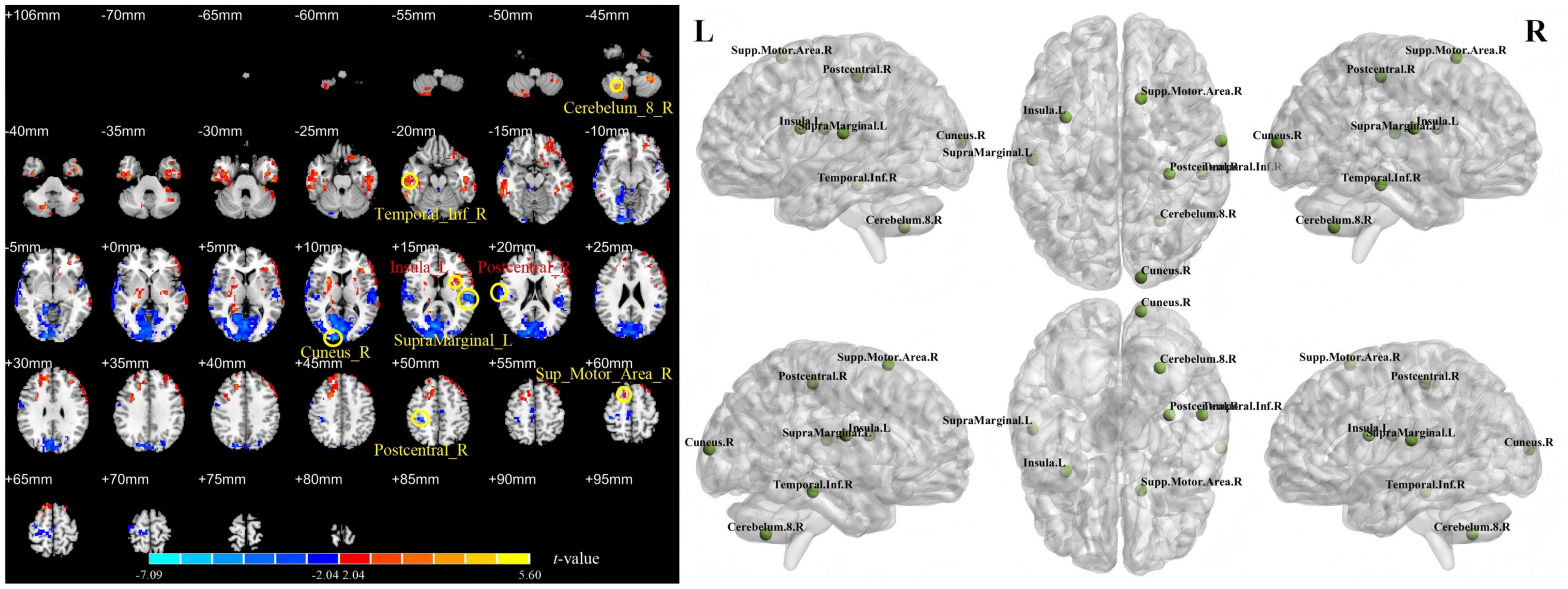
Differences in fALFF between stroke patients (11 in the rTMS group and 10 in the sham rTMS group) at baseline and healthy controls. Cerebellum_8_R = lobule VIII of right cerebellar hemisphere; Temporal_Inf_R = right inferior temporal gyrus; SupraMarginal_L = left supramarginal gyrus; Cuneus_R = right cuneus; Postcentral_R = right postcentral gyrus; Insula_L = left insula; Supp_Motor_Area_R = right supplementary motor area. Each named area refers to the region containing the voxel with the peak *t*-value (i.e., the greatest difference) within its respective cluster.

**Figure 4:**
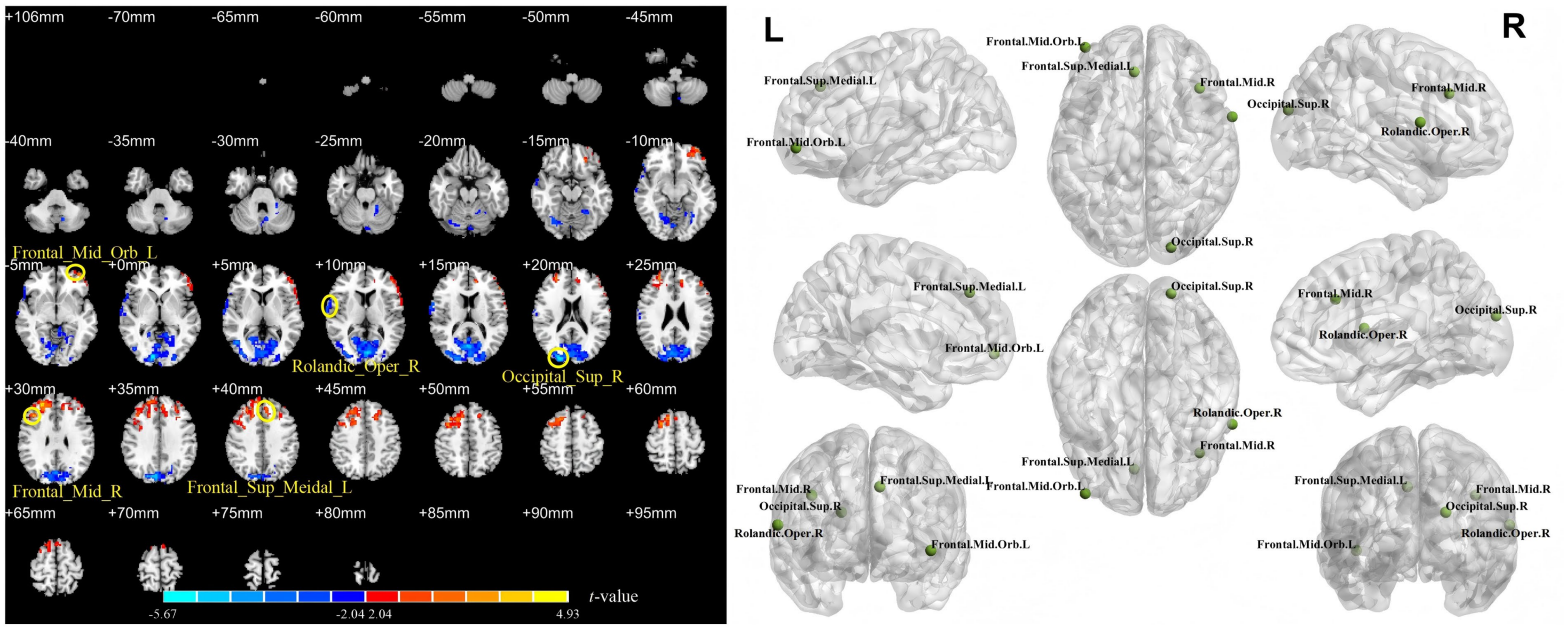
Differences in PerAF between stroke patients (11 in the rTMS group and 10 in the sham rTMS group) at baseline and healthy controls. Occipital_Sup_R = right superior occipital gyrus; Rolandic_Oper_R = right rolandic operculum; Frontal_Mid_Orb_L = left middle frontal gyrus, pars orbitalis; Frontal_Sup_Medial_L = left medial superior frontal gyrus; Frontal_Mid_R = right middle frontal gyrus. Each named area refers to the region containing the voxel with the peak *t*-value (i.e., the greatest difference) within its respective cluster.

Table 1 and Figures 2–4 show the brain regions with significant differences in ALFF, fALFF, or PerAF between patients at baseline (all patients combined) and healthy controls. Supplementary Figures 3–5 show the spatial patterns of the results with mean FD regressed out, which closely resemble those of the primary analysis. In addition, Supplementary Table 4 presents the significant clusters identified between pretreatment patients and healthy controls when applying more stringent correction criteria, including GRF correction with a voxel-level threshold of *p* < 0.01 and the false discovery rate (FDR) method.

**Figure 5:**
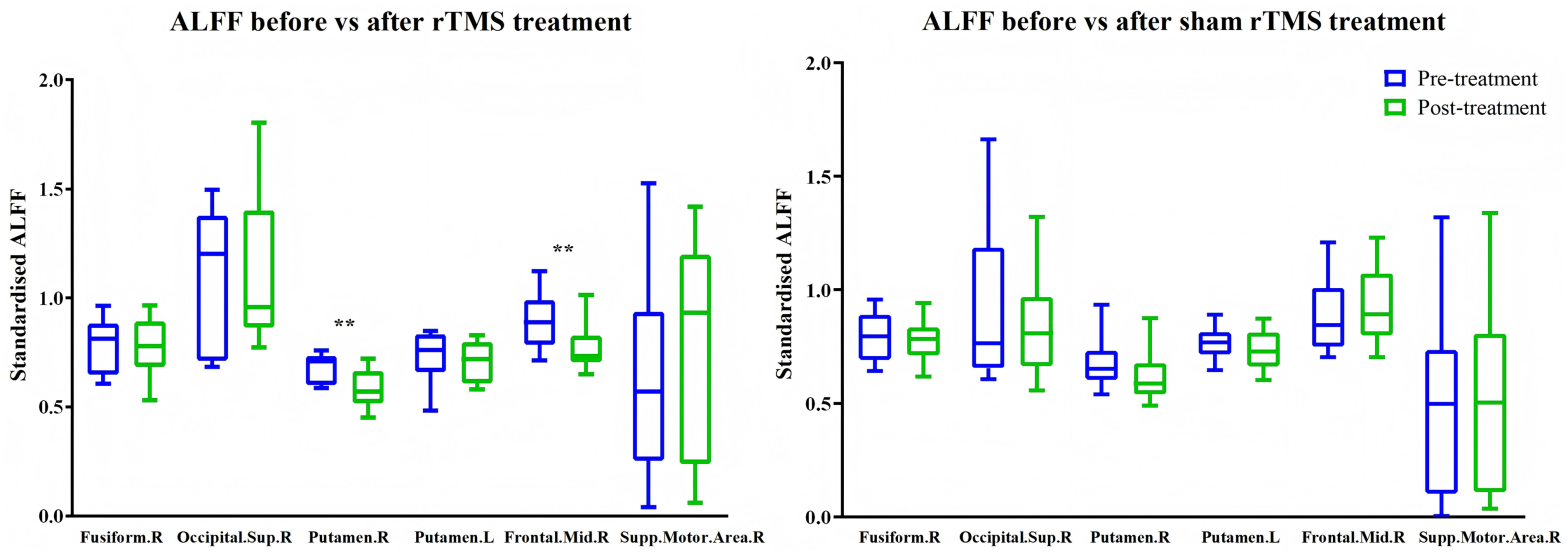
Effects of active (left) and sham rTMS (right) treatments on ALFF in brain areas that showed baseline differences between stroke patients and healthy controls. Fusiform.R = right fusiform gyrus; Occipital.Sup.R = right superior occipital gyrus; Putamen.R = right putamen; Putamen.L = left putamen; Frontal.Mid.R = right middle frontal gyrus; Supp.Motor.Area.R = right supplementary motor area. **p = 0.006 and 0.003 (not significant after Bonferroni correction).

**Figure 6:**
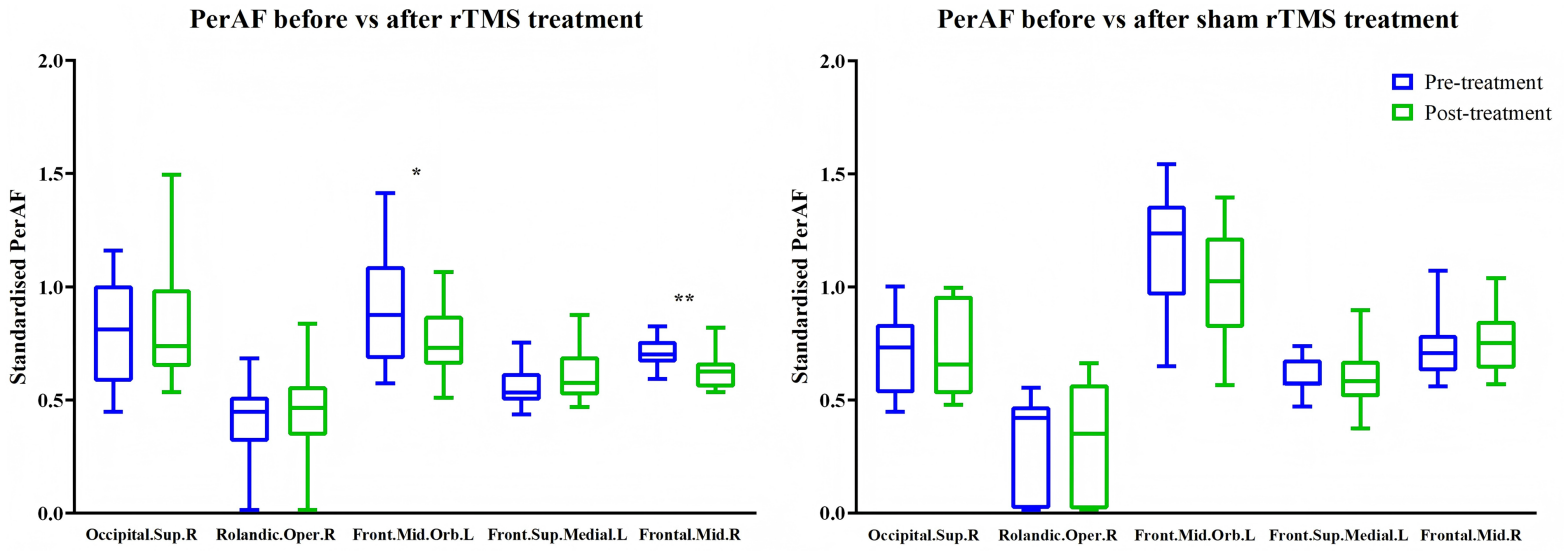
Effects of active (left) and sham rTMS (right) treatments on PerAF in brain areas that showed baseline differences between stroke patients and healthy controls. Occipital.Sup.R = right superior occipital gyrus; Rolandic.Oper.R = right rolandic operculum; Front.Mid.Orb.L = left middle frontal gyrus, pars orbitalis; Front.Sup.Medial.L = left medial superior frontal gyrus; Frontal.Mid.R = right middle frontal gyrus. *p = 0.047, **p = 0.008 (not significant after Bonferroni correction).

**Figure 7:**
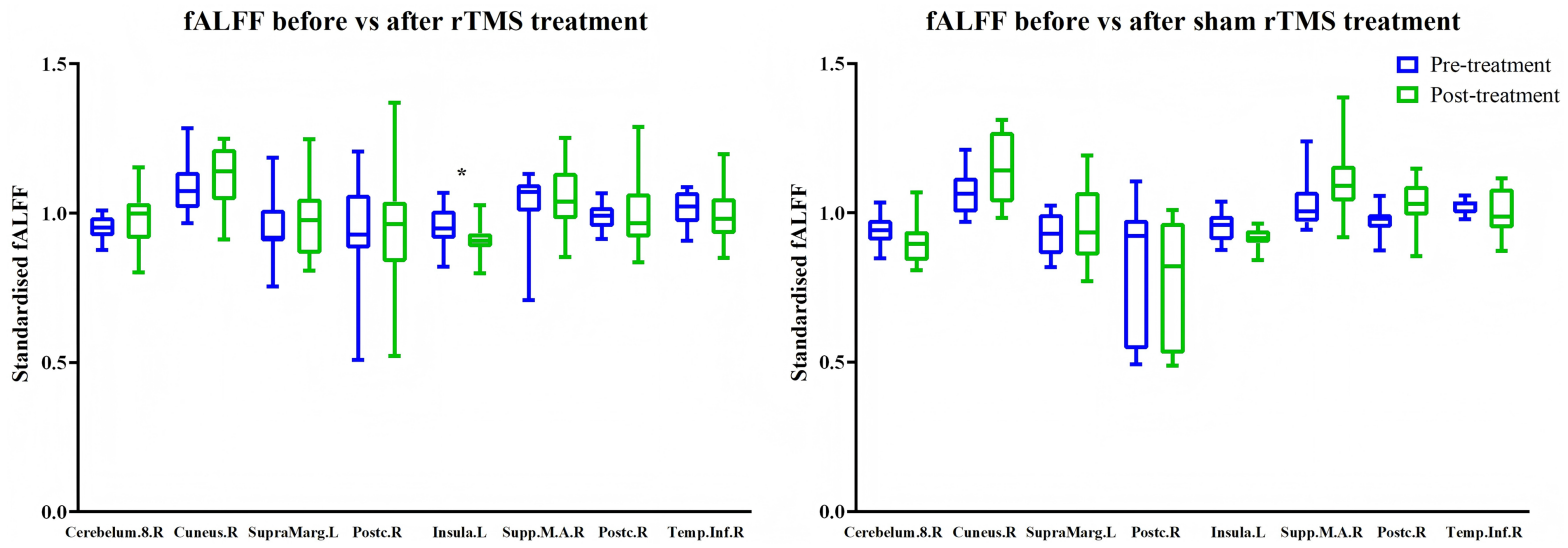
Effects of active (left) and sham rTMS (right) treatments on fALFF in brain areas that showed baseline differences between stroke patients and healthy controls. Cerebellum.8.R = lobule VIII of right cerebellar hemisphere; Cuneus.R = right cuneus; SupraMarg.L = left supramarginal gyrus; Postc.R = right postcentral gyrus; Insula.L = left insula; Supp.M.A.R = right supplementary motor area; Temp.Inf.R = right inferior temporal gyrus. *p = 0.049 (not significant after Bonferroni correction).

Repetitive TMS treatment reduced ALFF (Table 3 and Figure 5) and PerAF (Table 4 and Figure 6) in the right middle frontal gyrus (ALFF: *t* = 3.91, *p* = 0.003, *d_z_* = −1.18; PerAF: *t* = 3.29, *p* = 0.008, *d_z_* = −0.99). It also reduced ALFF in the right putamen (*t* = 3.48, *p* = 0.006, *d_z_* = −1.05), fALFF in the left insula (Table 5 and Figure 7; *t* = 2.25, *p* = 0.049, *d_z_* = −0.68), and PerAF in the left middle frontal gyrus, pars orbitalis (*t* = 2.26, *p* = 0.047, *d_z_* = −0.68). Cohen’s *d_z_* was calculated using the mean and standard deviation of the differences between pre- and post-treatment. However, these changes did not survive Bonferroni correction. In contrast, sham rTMS treatment had no effect on ALFF, fALFF, or PerAF (Tables 3–5 and Figures 5–7) in any of the brain regions listed in Table 1 before Bonferroni correction.

**Table 3:** ALFF before and after treatment in the rTMS and sham rTMS groups.

Outcome	Group	Brain region (aal)	Mean (SD)			^2^Statistics
			Pre-treatment	Post-treatment	^1^Difference	
ALFF	rTMS	Fusiform_R	0.78 (0.12)	0.78 (0.13)	0.003 (0.12)	*t* = 0.07, *p* = 0.948
		Occipital_Sup_R	1.08 (0.33)	1.13 (0.37)	0.06 (0.31)	*t* = 0.60, *p* = 0.563
		Putamen_L	0.73 (0.12)	0.71 (0.09)	−0.02 (0.17)	*t* = 0.37, *p* = 0.720
		Putamen_R	0.68 (0.07)	0.58 (0.09)	−0.09 (0.09)	*t = 3.48, *p* = 0.006
		Frontal_Mid_R	0.90 (0.13)	0.78 (0.12)	−0.12 (0.10)	*t = 3.91, *p* = 0.003
		Supp_Motor_Area_R	0.63 (0.46)	0.81 (0.47)	0.18 (0.67)	*t* = 0.89, *p* = 0.392
	Sham rTMS	Fusiform_R	0.80 (0.11)	0.78 (0.09)	−0.02 (0.12)	*t* = 0.54, *p* = 0.605
		Occipital_Sup_R	0.91 (0.35)	0.83 (0.23)	−0.08 (0.37)	*t* = 0.71, *p* = 0.499
		Putamen_L	0.77 (0.08)	0.73 (0.09)	−0.04 (0.12)	*t* = 0.90, *p* = 0.391
		Putamen_R	0.68 (0.11)	0.62 (0.11)	−0.06 (0.16)	*W* = −23, *p* = 0.275
		Frontal_Mid_R	0.89 (0.18)	0.93 (0.17)	0.04 (0.23)	*t* = 0.54, *p* = 0.603
		Supp_Motor_Area_R	0.48 (0.42)	0.53 (0.42)	0.05 (0.54)	*W* = 19, *p* = 0.375

1. Difference = Mean and standard deviation of the differences between post-treatment and pre-treatment values. 2. Calculated using paired *t*-test or Wilcoxon matched-pairs signed rank test. Fusiform_R = right fusiform; Occipital_Sup_R = right superior occipital gyrus; Putamen_L = left putamen; Putamen_R = right putamen; Frontal_Mid_R = right middle frontal gyrus; Supp_Motor_Area_R = right supplementary motor area. * Did not remain significant after Bonferroni correction.

**Table 4:** PerAF before and after treatment in the rTMS and sham rTMS groups.

Outcome	Group	Brain region (aal)	Mean (SD)			^2^Statistics
			Pre-treatment	Post-treatment	^1^Difference	
PerAF	rTMS	Occipital_Sup_R	0.82 (0.23)	0.85 (0.29)	0.04 (0.20)	*t* = 0.59, *p* = 0.569
		Rolandic_Oper_R	0.39 (0.21)	0.43 (0.24)	0.04 (0.14)	*t* = 0.96, *p* = 0.359
		Frontal_Mid_Orb_L	0.90 (0.26)	0.76 (0.16)	−0.14 (0.21)	*t = 2.26, *p* = 0.047
		Frontal_Sup_Medial_L	0.56 (0.09)	0.61 (0.12)	0.05 (0.13)	*t* = 1.21, *p* = 0.253
		Frontal_Mid_R	0.71 (0.07)	0.64 (0.09)	−0.08 (0.08)	*t = 3.29, *p* = 0.008
	Sham rTMS	Occipital_Sup_R	0.72 (0.17)	0.72 (0.21)	0.01 (0.24)	*t* = 0.11, *p* = 0.911
		Rolandic_Oper_R	0.29 (0.24)	0.30 (0.26)	0.01 (0.12)	*t* = 0.35, *p* = 0.734
		Frontal_Mid_Orb_L	1.15 (0.27)	1.02 (0.25)	−0.13 (0.19)	*t* = 2.13, *p* = 0.062
		Frontal_Sup_Medial_L	0.60 (0.08)	0.60 (0.15)	−0.002 (0.15)	*t* = 0.05, *p* = 0.965
		Frontal_Mid_R	0.73 (0.15)	0.76 (0.15)	0.03 (0.20)	*t* = 0.41, *p* = 0.689

1. Difference = Mean and standard deviation of the differences between post-treatment and pre-treatment values. 2. Calculated using paired *t*-test. Occipital_Sup_R = right superior occipital gyrus; Rolandic_Oper_R = right rolandic operculum; Frontal_Mid_Orb_L = left middle frontal gyrus, pars orbitalis; Frontal_Sup_Medial_L = left medial superior frontal gyrus Frontal_Mid_R = right middle frontal gyrus. * Did not remain significant after Bonferroni correction.

**Table 5:** fALFF before and after treatment in the rTMS and sham rTMS groups.

Outcome	Group	Brain region (aal)	Mean (SD)			^2^Statistics
			Pre-treatment	Post-treatment	^1^Difference	
fALFF	rTMS	Cerebellum_8_R	0.95 (0.05)	0.98 (0.09)	0.03 (0.12)	*t* = 0.91, *p* = 0.384
		Temporal_Inf_R	1.02 (0.06)	1.00 (0.11)	−0.02 (0.14)	*t* = 0.50, *p* = 0.625
		SupraMarginal_L	0.95 (0.11)	0.97 (0.12)	0.02 (0.13)	*W* = 0, *p* > 0.999
		Cuneus_R	1.06 (0.10)	1.07 (0.11)	0.01 (0.13)	*t* = 1.12, *p* = 0.288
		Postcentral_R	0.92 (0.22)	0.93 (0.23)	0.004 (0.13)	*t* = 0.11, *p* = 0.916
		Insula_L	0.96 (0.07)	0.91 (0.06)	−0.05 (0.07)	*t = 2.25, *p* = 0.049
		Supp_Motor_Area_R	1.03 (0.12)	1.06 (0.12)	0.03 (0.14)	*t* = 0.73, *p* = 0.483
		Postcentral_R	0.99 (0.05)	1.00 (0.12)	0.01 (0.11)	*t* = 0.34, *p* = 0.738
	Sham rTMS	Cerebellum_8_R	0.94 (0.05)	0.90 (0.08)	−0.04 (0.09)	*t* = 1.29, *p* = 0.230
		Temporal_Inf_R	1.02 (0.03)	1.00 (0.03)	−0.02 (0.09)	*t* = 0.80, *p* = 0.443
		SupraMarginal_L	0.93 (0.07)	0.96 (0.14)	0.03 (0.11)	*t* = 0.89, *p* = 0.395
		Cuneus_R	1.07 (0.07)	1.15 (0.13)	0.08 (0.12)	*t* = 2.08, *p* = 0.068
		Postcentral_R	0.80 (0.24)	0.77 (0.21)	−0.03 (0.07)	*t* = 1.37, *p* = 0.203
		Insula_L	0.95 (0.05)	0.92 (0.04)	−0.04 (0.06)	*t* = 2.06, *p* = 0.069
		Supp_Motor_Area_R	1.03 (0.09)	1.11 (0.13)	0.08 (0.13)	*t* = 1.93, *p* = 0.085
		Postcentral_R	0.97 (0.05)	1.03 (0.08)	0.06 (0.09)	*t* = 2.00, *p* = 0.077

1. Difference = Mean and standard deviation of the differences between post-treatment and pre-treatment values. 2. Calculated using paired *t*-test or Wilcoxon matched-pairs signed rank test. Cerebellum_8_R = lobule VIII of right cerebellar hemisphere; Temporal_Inf_R = right inferior temporal gyrus; SupraMarginal_L = left supramarginal gyrus; Cuneus_R = right cuneus; Postcentral_R = right postcentral gyrus; Insula_L = left insula; Supp_Motor_Area_R = right supplementary motor area. * Did not remain significant after Bonferroni correction.

### 3.3 Graph theory analysis and reproducibility analysis

Graph theory analysis revealed a significant decrease in the clustering coefficient (Table 6; *t* = −4.71, *p* = 0.0008, *d_z_* = −1.42) and local efficiency (Table 7; *t* = −4.31, *p* = 0.0015, *d_z_* = −1.36) in the left medial superior frontal gyrus after rTMS treatment. However, neither survived Bonferroni correction. No significant changes were found following rTMS in the other nodal graph metrics or in the global graph metrics, including small-world properties (*σ*, *γ*, *λ*, Cp, Lp) and network efficiency (Eg, Eloc). Sham rTMS did not significantly affect any of the graph metrics when comparing pre- and post-treatment data. Within the defined range of sparsity thresholds, the functional brain networks of both patient groups exhibited small-world topology both before and after treatment (Supplementary Figures 1, 2).

**Table 6:** Decrease in clustering coefficient of the left medial superior frontal gyrus after rTMS treatment (proportional-density window: 0.05–0.5; positive functional connectivity matrix).

Metric	Group	Brain region	Mean (SD)		Test statistic
			Pre-treatment	Post-treatment	
Node – Clustering Coefficient	rTMS	Fusiform_R	0.07 (0.05)	0.11 (0.08)	*t* = 1.29, *p* = 0.22
		Occipital_Sup_R_1	0.18 (0.08)	0.19 (0.07)	*t* = 0.64, *p* = 0.54
		Putamen_L	0.11 (0.08)	0.07 (0.03)	*t* = −1.67, *p* = 0.13
		Putamen_R	0.13 (0.10)	0.07 (0.05)	*t* = −1.55, *p* = 0.15
		Frontal_Mid_R	0.14 (0.05)	0.07 (0.04)	*t* = −3.10, *p* = 0.01
		Supp_Motor_Area_R_1	0.08 (0.05)	0.10 (0.05)	*t* = 0.85, *p* = 0.41
		Cerebellum_8_R	0.08 (0.04)	0.08 (0.07)	*t* = −0.07, *p* = 0.95
		Temporal_Inf_R	0.10 (0.05)	0.11 (0.07)	*t* = 0.50, *p* = 0.63
		SupraMarginal_L	0.06 (0.03)	0.11 (0.07)	*t* = 2.70, *p* = 0.02
		Cuneus_R	0.16 (0.06)	0.15 (0.07)	*t* = −0.55, *p* = 0.59
		Postcentral_R_1	0.07 (0.03)	0.12 (0.06)	*t* = 2.11, *p* = 0.06
		Insula_L	0.09 (0.07)	0.07 (0.02)	*t* = −0.71, *p* = 0.49
		Supp_Motor_Area_R_2	0.10 (0.05)	0.10 (0.04)	*t* = −0.17, *p* = 0.87
		Postcentral_R_2	0.10 (0.06)	0.10 (0.06)	*t* = 0.11, *p* = 0.92
		Occipital_Sup_R_2	0.16 (0.06)	0.16 (0.06)	*t* = 0.11, *p* = 0.92
		Rolandic_Oper_R	0.09 (0.04)	0.12 (0.07)	*t* = 1.13, *p* = 0.29
		Frontal_Mid_Orb_L	0.11 (0.05)	0.08 (0.06)	*t* = −1.63, *p* = 0.13
		Frontal_Sup_Medial_L	0.15 (0.05)	0.08 (0.04)	*t = −4.71, *p* = 0.0008
Node - Clustering Coefficient	Sham rTMS	Fusiform_R	0.08 (0.06)	0.09 (0.07)	*t* = 0.55, *p* = 0.60
		Occipital_Sup_R_1	0.17 (0.05)	0.19 (0.05)	*t* = 1.18, *p* = 0.27
		Putamen_L	0.10 (0.07)	0.09 (0.07)	*t* = −0.92, *p* = 0.38
		Putamen_R	0.07 (0.05)	0.08 (0.08)	*t* = 0.35, *p* = 0.73
		Frontal_Mid_R	0.10 (0.05)	0.12 (0.05)	*t* = 1.01, *p* = 0.34
		Supp_Motor_Area_R_1	0.10 (0.08)	0.09 (0.05)	*t* = −0.24, *p* = 0.82
		Cerebellum_8_R	0.09 (0.07)	0.04 (0.04)	*t* = −1.49, *p* = 0.17
		Temporal_Inf_R	0.11 (0.07)	0.13 (0.06)	*t* = 0.44, *p* = 0.67
		SupraMarginal_L	0.09 (0.06)	0.07 (0.06)	*t* = −0.89, *p* = 0.40
		Cuneus_R	0.15 (0.06)	0.16 (0.07)	*t* = 0.35, *p* = 0.74
		Postcentral_R_1	0.08 (0.04)	0.08 (0.07)	*t* = 0.10, *p* = 0.93
		Insula_L	0.06 (0.06)	0.04 (0.04)	*t* = −1.12, *p* = 0.29
		Supp_Motor_Area_R_2	0.11 (0.08)	0.11 (0.05)	*t* = 0.08, *p* = 0.94
		Postcentral_R_2	0.12 (0.06)	0.13 (0.05)	*t* = 0.36, *p* = 0.73
		Occipital_Sup_R_2	0.17 (0.07)	0.19 (0.05)	*t* = 0.70, *p* = 0.50
		Rolandic_Oper_R	0.10 (0.05)	0.10 (0.07)	*t* = 0.04, *p* = 0.97
		Frontal_Mid_Orb_L	0.11 (0.10)	0.11 (0.07)	*t* = −0.12, *p* = 0.90
		Frontal_Sup_Medial_L	0.11 (0.08)	0.10 (0.05)	*t* = −0.40, *p* = 0.70

Area under the curve was used for statistical analysis of this graph metric. * Not significant after Bonferroni correction for multiple comparisons. Frontal_Sup_Medial_L = left medial superior frontal gyrus.

**Table 7:** Decrease in local efficiency of the left medial superior frontal gyrus after rTMS treatment (proportional-density window: 0.05–0.5; positive functional connectivity matrix).

Metric	Group	Brain region	Pre-treatment	Post-treatment	Test statistic
Node – Local efficiency	rTMS	Fusiform_R	0.08 (0.06)	0.12 (0.08)	*t* = 1.18, *p* = 0.27
		Occipital_Sup_R_1	0.17 (0.08)	0.18 (0.06)	*t* = 0.72, *p* = 0.49
		Putamen_L	0.13 (0.08)	0.09 (0.04)	*t* = −1.75, *p* = 0.11
		Putamen_R	0.14 (0.10)	0.09 (0.06)	*t* = −1.34, *p* = 0.21
		Frontal_Mid_R	0.15 (0.05)	0.09 (0.04)	*t* = −3.19, *p* = 0.01
		Supp_Motor_Area_R_1	0.10 (0.05)	0.13 (0.06)	*t* = 1.35, *p* = 0.21
		Cerebellum_8_R	0.11 (0.04)	0.10 (0.08)	*t* = −0.43, *p* = 0.68
		Temporal_Inf_R	0.11 (0.04)	0.12 (0.08)	*t* = 0.40, *p* = 0.70
		SupraMarginal_L	0.08 (0.04)	0.14 (0.08)	*t* = 2.76, *p* = 0.02
		Cuneus_R	0.18 (0.06)	0.17 (0.07)	*t* = −0.93, *p* = 0.37
		Postcentral_R_1	0.10 (0.04)	0.13 (0.06)	*t* = 1.67, *p* = 0.13
		Insula_L	0.11 (0.08)	0.08 (0.02)	*t* = −1.04, *p* = 0.32
		Supp_Motor_Area_R_2	0.11 (0.05)	0.12 (0.05)	*t* = 0.77, *p* = 0.46
		Postcentral_R_2	0.12 (0.06)	0.12 (0.06)	*t* = −0.07, *p* = 0.95
		Occipital_Sup_R_2	0.16 (0.07)	0.17 (0.05)	*t* = 0.34, *p* = 0.74
		Rolandic_Oper_R	0.11 (0.05)	0.13 (0.06)	*t* = 0.80, *p* = 0.44
		Frontal_Mid_Orb_L	0.13 (0.05)	0.10 (0.08)	*t* = −1.07, *p* = 0.31
		Frontal_Sup_Medial_L	0.16 (0.05)	0.10 (0.04)	*t = −4.31, *p* = 0.0015
Node – Local efficiency	Sham rTMS	Fusiform_R	0.09 (0.06)	0.10 (0.07)	*t* = 0.46, *p* = 0.66
		Occipital_Sup_R_1	0.16 (0.06)	0.18 (0.05)	*t* = 0.93, *p* = 0.38
		Putamen_L	0.12 (0.07)	0.10 (0.08)	*t* = −0.90, *p* = 0.39
		Putamen_R	0.08 (0.05)	0.09 (0.08)	*t* = 0.19, *p* = 0.86
		Frontal_Mid_R	0.11 (0.04)	0.14 (0.05)	*t* = 1.61, *p* = 0.14
		Supp_Motor_Area_R_1	0.11 (0.07)	0.11 (0.05)	*t* = −0.11, *p* = 0.92
		Cerebellum_8_R	0.11 (0.08)	0.06 (0.05)	*t* = −1.45, *p* = 0.18
		Temporal_Inf_R	0.13 (0.07)	0.15 (0.06)	*t* = 0.78, *p* = 0.46
		SupraMarginal_L	0.10 (0.05)	0.09 (0.06)	*t* = −0.50, *p* = 0.63
		Cuneus_R	0.17 (0.05)	0.18 (0.06)	*t* = 0.42, *p* = 0.68
		Postcentral_R_1	0.09 (0.05)	0.09 (0.08)	*t* = 0.20, *p* = 0.85
		Insula_L	0.07 (0.07)	0.04 (0.04)	*t* = −1.35, *p* = 0.21
		Supp_Motor_Area_R_2	0.12 (0.08)	0.13 (0.05)	*t* = 0.48, *p* = 0.64
		Postcentral_R_2	0.14 (0.04)	0.16 (0.04)	*t* = 1.04, *p* = 0.33
		Occipital_Sup_R_2	0.16 (0.07)	0.18 (0.05)	*t* = 0.72, *p* = 0.49
		Rolandic_Oper_R	0.11 (0.06)	0.12 (0.07)	*t* = 0.29, *p* = 0.78
		Frontal_Mid_Orb_L	0.12 (0.09)	0.13 (0.07)	*t* = 0.36, *p* = 0.73
		Frontal_Sup_Medial_L	0.12 (0.07)	0.12 (0.04)	*t* = −0.17, *p* = 0.87

Area under the curve was used for statistical analysis of this graph metric. * Not significant after Bonferroni correction for multiple comparisons. Frontal_Sup_Medial_L = left medial superior frontal gyrus.

The results of the reproducibility analysis were consistent with the original findings, showing that rTMS treatment significantly decreased the nodal clustering coefficient (Supplementary Table 1; *t* = −4.60, *p* = 0.001, *d_z_* = −1.39) and local efficiency (Supplementary Table 2; *t* = −4.41, *p* = 0.0013, *d_z_* = −1.33) in the left medial superior frontal gyrus. In addition, significant reductions in clustering coefficient (Supplementary Table 1; *t* = −4.03, *p* = 0.0024, *d_z_* = −1.21) and local efficiency (Supplementary Table 2; *t* = −3.95, *p* = 0.0027, *d_z_* = −1.19) were found in the right middle frontal gyrus, along with an increase in betweenness centrality (Supplementary Table 3; *t* = 4.13, *p* = 0.0021, *d_z_* = 1.24). However, these effects also did not survive Bonferroni correction. No other significant changes in graph metrics were observed following rTMS, and sham stimulation produced no significant pre–post effects.

### 3.4 Correlational analysis

In the rTMS group, the correlation between reductions in SSA scores and decreased local efficiency (*r* = −0.64, *p* = 0.034) in the left medial superior frontal gyrus did not survive Bonferroni correction. No other significant correlations were found between changes (post- minus pre-intervention) in rs-fMRI data and clinical ratings.

### 3.5 Linear regression

Simple linear regression showed that the post-treatment delay interval (days) did not significantly predict changes in EAT-10, SSA or MMASA scores, nor did it predict changes in regional activity or graph metrics, before Bonferroni correction. These findings suggest that a longer delay between rTMS treatment and post-treatment assessment may not necessarily lead to greater changes in clinical scores or neuroimaging outcomes.

## 4 Discussion

In this study, we first compared dysphagic stroke patients with healthy controls to identify brain regions exhibiting abnormal ALFF, fALFF, or PerAF in patients prior to intervention. We then investigated whether a course of active or sham rTMS treatment modified the amplitude of spontaneous fluctuations in these abnormal brain regions, and whether it led to changes in the topological properties of the network formed by these regions. Finally, we examined the correlations between changes in clinical ratings and brain function. We found that 2 weeks of 5 Hz rTMS targeting the contralesional mylohyoid motor cortical representation reduced ALFF and PerAF in the right (contralesional) middle frontal gyrus, ALFF in the right putamen, fALFF in the left insula, and PerAF in the left middle frontal gyrus (pars orbitalis). rTMS also reduced local efficiency and clustering coefficient in the left medial superior frontal gyrus. However, these changes did not survive Bonferroni correction, possibly due to low statistical power (*post hoc* power estimates for ALFF, fALFF, PerAF, clustering coefficient and local efficiency were ≤ 0.51). Nonetheless, the findings may still be meaningful, as (1) the associated effect sizes were medium to large (ALFF: *d_z_* = −1.18 and −1.05; fALFF: *d_z_* = −0.68; PerAF: *d_z_* = −0.99 and −0.68; clustering coefficient: *d_z_* = −1.42; local efficiency: *d_z_* = −1.36), indicating noticeable differences in the neural activity metrics following rTMS; and (2) no comparable changes were observed in patients receiving sham rTMS, even before correction.

The dysphagic stroke patients in our study suffered lesions in various locations (including the cortex, subcortical structures, or brainstem), and their duration of illness at baseline ranged from 10 to 70 days, resulting in a heterogeneous sample. The brain areas shown to have different ALFF, fALFF, or PerAF in patients compared to healthy controls were broadly consistent with the regions involved in the swallowing network (Cheng et al., 2022). Specifically, we found increased amplitude of spontaneous oscillations in the left medial superior frontal gyrus, middle frontal gyrus (pars orbitalis), and insula, as well as in the right middle frontal gyrus, supplementary motor area, fusiform gyrus, inferior temporal gyrus, cerebellar lobule VIII, and bilateral putamen. In contrast, we observed decreased amplitude in the left supramarginal gyrus and in the right superior occipital gyrus, supplementary motor area, postcentral gyrus, rolandic operculum, and cuneus. Several of these abnormal brain regions—such as the lentiform nucleus (containing the putamen), insula, supramarginal gyrus, middle frontal gyrus, postcentral gyrus, visual cortex, inferior temporal gyrus, and cerebellum—have also been reported in previous rs-fMRI studies comparing ALFF and/or fALFF between post-stroke dysphagic patients and healthy controls (Jiao et al., 2020; Long et al., 2019; Quan et al., 2022).

Our stimulation protocol was developed based on the idea of contralesional hemisphere compensation, which proposes that the uninjured hemisphere may assume lost functions when one hemisphere is severely damaged (Wang et al., 2020). This model of stroke rehabilitation is supported by studies showing that increased cortical excitability in the uninjured hemisphere of dysphagic stroke patients was associated with recovery of swallowing function (Fraser et al., 2002; Hamdy et al., 1998). Patients in Hamdy et al. (1998) had, on average, severe stroke impairment at baseline, while those in Fraser et al. (2002) presented with a range of stroke severities. However, our results did not show the increased functional engagement in the contralesional (i.e., uninjured) hemisphere that is typically associated with functional compensation following high-frequency rTMS to the uninjured hemisphere. On the contrary, we observed a decrease in activity in the contralesional middle frontal gyrus and putamen, which may be better explained within the framework of the bi-hemispheric balance model of stroke recovery. This model is also evidence-based (Casula et al., 2021; Murase et al., 2004). It proposes that, in healthy people, the two motor cortices exert mutual interhemispheric inhibition during unilateral movements, maintaining a dynamic balance. After a unilateral stroke, the interhemispheric balance is disrupted. The lesioned hemisphere may weaken inhibitory control over the uninjured side, while the uninjured hemisphere may become hyperexcitable and exert excessive inhibition on the lesioned side, potentially hindering motor recovery (Alia et al., 2017; Casula et al., 2021; Murase et al., 2004). Notably, patients in our study showed increased baseline activity in the right middle frontal gyrus and putamen compared to healthy controls, which was decreased by the rTMS intervention. Within the context of the bi-hemispheric balance model, this effect of rTMS might reflect suppression of maladaptive contralesional overactivity, potentially contributing to a restoration of interhemispheric balance. However, it remains uncertain whether increased contralesional activity after stroke is beneficial or maladaptive. Our interpretation is therefore speculative, particularly given the lack of significant correlations between reductions in right middle frontal and putamen activity and behavioral improvement.

Moreover, we observed that elevated baseline activity in the left (ipsilesional) insula and middle frontal gyrus (pars orbitalis) was reduced following rTMS. According to the bi-hemispheric balance theory, suppressing overactivity in the contralesional hemisphere is expected to enhance activity in the lesioned hemisphere, thereby facilitating stroke recovery. However, our results showed reduced activity in the lesioned hemisphere, suggesting that this finding may not be fully explained by the theory. Previous evidence indicates that inhibiting the lesioned hemisphere can also facilitate recovery. For instance, Di Lazzaro et al. (2013) applied inhibitory continuous theta-burst stimulation (cTBS) over the lesioned hemisphere in chronic stroke patients undergoing physical rehabilitation and found that real cTBS achieved better functional outcomes than sham stimulation. The authors speculated that the additional effect of TMS beyond physical therapy might be attributed to homeostatic plasticity. That is, by reducing cortical excitability in the lesioned hemisphere, cTBS may have “primed” the brain to become more responsive to motor training—enhancing its capacity for learning-related potentiation, rather than directly improving motor behavior. Similarly, the reduced activity in the ipsilesional insular and middle frontal gyrus (pars orbitalis) that we observed may have contributed to functional improvement. These findings illustrate the complexity of the processes driving post-stroke recovery.

We also found that rTMS decreased the nodal clustering coefficient and local efficiency in the left medial superior frontal gyrus. Both graph metrics reflect the efficiency of communication within the region’s immediate network. Thus, this may indicate a reduction in functional connectivity among neighboring nodes of the left medial superior frontal gyrus, leading to diminished local communication. One possible explanation is that rTMS may have induced synaptic pruning (Tang et al., 2021), a process in which weak or unused synapses are eliminated and frequently used ones are strengthened through activity-dependent mechanisms. This refinement may improve network efficiency and support better motor performance (Tang et al., 2018). In our case, it is possible that rTMS weakened connections around the ipsilesional medial superior frontal gyrus. The reproducibility analysis suggests that synaptic pruning may also have occurred in the right middle frontal gyrus. The decrease in clustering coefficient and local efficiency, accompanied by an increase in betweenness centrality, implies that rTMS may have weakened functional connectivity among the region’s immediate neighbors, although the effect sizes were slightly smaller than in the left medial superior frontal gyrus. Meanwhile, the right middle frontal gyrus itself may have assumed a more integrative or ‘bridging’ role in facilitating communication within the functional network, perhaps through synaptic pruning-related reshaping of network topology (Oldham and Fornito, 2019).

The insula and middle frontal gyrus are activated both during swallowing and prior to the onset of volitional swallowing (Hamdy, 2006; Jing et al., 2020). The insula is among the brain regions most consistently activated during swallowing (Cheng et al., 2022; Hamdy, 2006). It is connected to sensorimotor areas critical for swallowing, such as the primary motor and premotor cortices, gustatory and olfactory structures, and the thalamus (Hamdy, 2006; Saito et al., 2016). The anterior portion of the insula is thought to receive, integrate and relay sensory inputs from the oral cavity to help trigger the motor command initiating swallowing (Hamdy, 2006; Saito et al., 2016). Thus, the insula may function as a sensory hub that contributes to integrating sensory signals (e.g., taste, smell, vision) with motor commands for the initiation and execution of swallowing (Cheng et al., 2022; Saito et al., 2016). In line with these observations, research on the neural correlates of dysphagic stroke suggests that lesions involving the insula, particularly the anterior insula, are strongly associated with dysphagia in both the short and long term. Supporting evidence includes a case study showing that patients with lesions confined to the insular cortex exhibited dysphagia only when the lesion was located in the anterior insula, whether assessed within 1 week or 4 months after stroke (Daniels and Foundas, 1997); an imaging study reporting that gray matter atrophy predominantly affecting the left anterior insula was more pronounced in dysphagic patients than in non-dysphagic patients and healthy controls (Guo et al., 2025); and a voxel-based lesion-symptom mapping analysis demonstrating that more extensive insular damage—with the center of maximum overlap in the anterior insula—was associated with poorer recovery of normal oral intake ≥4 weeks after stroke (Galovic et al., 2017).

The middle frontal gyrus may be particularly involved in the cognitive aspects of swallowing planning or preparation. Lesions in this region have been associated with swallowing hesitation, characterized by a prolonged delay before initiating the swallow (Saito et al., 2016). Recent evidence also indicates that the middle frontal gyrus participates in both early (sensory detection) and late (cognitive access) phases of awareness, suggesting a role in bridging perception and conscious decision-making (Fang et al., 2024). Moreover, it is activated during both the execution and mental imagery of swallowing as part of a broader swallowing network—most consistently involving the precentral and postcentral gyri and the insula—and appears to be more frequently recruited during consciously controlled swallows than during spontaneous, automatic swallows (Kober et al., 2019; Martin et al., 2001). Relatively few studies have examined the role of the middle frontal gyrus in dysphagic stroke. Our previous work (Zeng et al., 2023) showed that dysphagic stroke patients exhibited higher fALFF in the right middle frontal gyrus in the slow-5 frequency band (0.01–0.027 Hz) compared to healthy controls. Huang et al. (2018) reported that, after receiving either traditional swallowing therapy or combined therapy with neuromuscular electrical stimulation to the anterior neck, patients with hemispheric (supratentorial) stroke showed increased functional connectivity between the right middle frontal cortex and the rest of the dorsal default mode network, as identified by independent component analysis. These findings suggest that middle frontal activity may be abnormal in dysphagic stroke and that its neuroplasticity is modifiable through treatment. In the present study, rTMS over the primary motor cortex reduced spontaneous activity in both the insula and middle frontal gyrus. The middle frontal gyrus may therefore act as an association area between the insula (a sensory center) and the primary motor cortex (a motor center) (Saito et al., 2016). Unlike the insula, which integrates and relays sensory information for motor initiation and execution, the middle frontal gyrus may help coordinate sensory and motor processes, perhaps through top-down control of voluntary behavior (Mars and Grol, 2007). Taken together, the observed rTMS effects on the insula align with existing evidence that proper insular function is critical for swallowing recovery. The contribution of the middle frontal gyrus to dysphagia and recovery is less well understood, but its involvement in consciously controlled swallowing suggests that modulation of this region could also support swallowing recovery.

The putamen is part of a motor cortico–basal ganglia–thalamo–cortical loop relevant to the pathophysiology of movement (Obeso et al., 2002). The posterior putamen, in particular, receives input from motor and somatosensory cortices—including the primary motor and premotor cortices, supplementary motor area, and primary somatosensory cortex—and communicates with the thalamus and brain stem via basal ganglia output nuclei (Obeso et al., 2002). Thus, the insula, putamen and middle frontal gyrus may belong to an extended sensorimotor network involved in swallowing. Moreover, the putamen may contribute to motor sequencing, with the anterior and posterior portions playing distinct roles: the anterior putamen may support accurate learning of sequences, while the posterior putamen may be more engaged in the automatic execution of well-learned sequences (Lehéricy et al., 2005). The posterior putamen has also been found to be recruited when constructing motor sequences from working memory (Menon et al., 2000). Meanwhile, the roles of the middle frontal gyrus (pars orbitalis) and medial superior frontal gyrus in post-stroke dysphagia are unclear. The pars orbitalis is typically associated with the inferior, rather than middle, frontal gyrus and has been implicated in cognitive functions such as language production and the perception of semantics and emotion (Ardila et al., 2017; Belyk et al., 2017; Nair et al., 2015). The medial superior frontal gyrus (also termed the medial frontal gyrus) forms the medial surface of the superior frontal gyrus and is often considered to contain the supplementary motor area (SMA) posteriorly (Zhang et al., 2012; Gaillard et al., 2017). However, the AAL atlas distinguishes the medial superior frontal gyrus and SMA as separate labels, so the former cannot be assumed to include the SMA. The functions of the AAL-defined medial superior frontal gyrus (Frontal_Sup_Medial) have not been clearly characterized. In healthy adults, activation in the medial frontal gyrus (used interchangeably with “SMA” in that study) during swallow execution increased with more effortful and complex motor control (Peck et al., 2010). Similarly, in acutely dysphagic stroke patients, SMA activation occurred during effortful swallowing—when participants were instructed to squeeze the back of the tongue and swallow as hard as possible—but not during normal swallowing before rehabilitation (Gu et al., 2024). Activation also re-emerged during normal swallowing after treatment, suggesting that SMA dysfunction may recover through rehabilitation (Gu et al., 2024). Although evidence for the medial frontal gyrus outside the SMA (i.e., the AAL-defined medial superior frontal gyrus) is limited, current findings suggest that this area may also contribute to swallowing impairment and recovery. Overall, our results indicate reduced activity and reorganized network topology within sensorimotor regions, implying that rTMS may have modulated both cortical and subcortical components of the sensorimotor network.

Few studies have utilized functional neuroimaging to explore the mechanisms underlying rTMS effects on post-stroke dysphagia. The only such study identified (Liu H. et al., 2022) applied 5 Hz rTMS over the ipsilesional mylohyoid cortical area of patients with unilateral or bilateral subcortical stroke. For patients with bilateral lesions, stimulation was delivered to the hemisphere with more extensive damage or to the side corresponding to the more severely affected limb. The intervention comprised 10 rTMS sessions over 2 weeks, each followed by traditional dysphagia therapy. Cerebral hemodynamic changes during swallowing, measured using functional near-infrared spectroscopy, were evaluated 2 weeks post-intervention. Compared with the sham group, rTMS-treated patients exhibited greater clinical improvement. After rTMS, swallowing elicited increased activation in the right prefrontal and motor cortices in patients with right hemispheric lesions relative to baseline, whereas no significant changes were observed following left-hemisphere stimulation in patients with left hemispheric lesions. Although both Liu H. et al. (2022) and the present study demonstrated clinical improvement, different rTMS protocols produced distinct effects on brain activity. Liu H. et al. (2022) applied high-frequency rTMS to the *lesioned* hemisphere and observed increased activation in that hemisphere, consistent with the bi-hemispheric balance model of post-stroke recovery. In contrast, the present study found that high-frequency rTMS over the *uninjured* hemisphere led to reduced activity in several cortical and subcortical areas (insula, middle frontal gyrus, and putamen). Direct comparison of rTMS effects on regional brain activity across studies remains difficult, given the scarcity of relevant neuroimaging research and the heterogeneity in stimulation parameters (e.g., site, intensity, frequency, duration) and assessment timing. Nevertheless, Dong et al. (2022) delivered 10 Hz rTMS (100% RMT, 250 pulses) to the dominant cerebellar hemisphere representation of the suprahyoid (swallowing) muscles in healthy young adults using a circular coil, and found increased ALFF in the cerebellum and brainstem, accompanied by decreased ALFF/ReHo (regional homogeneity) in the superior temporal lobe, insula, putamen and supplementary motor area. The authors interpreted this pattern—cortical suppression alongside cerebellar and brainstem enhancement—as reflecting facilitation of automatic swallowing circuits while inhibiting or down-regulating cortical regions involved in voluntary swallowing control. The findings of Liu H. et al. (2022) and Dong et al. (2022) therefore support the notion that the rTMS-induced changes observed in the frontal, insular and putaminal regions in the current study likely represent modulation of swallowing-related areas rather than arbitrary fluctuations.

## 5 Limitations and future research

First, this exploratory study had a relatively small sample size (11 rTMS-treated patients, 10 sham-treated patients, and 14 healthy controls), which limited statistical power. The *post hoc* power estimates for pre–post changes in the neural metrics were below 60% at Bonferroni-corrected *α* levels; at the uncorrected *α* level of 0.05, the corresponding values ranged from 0.53 to 0.99. Given the medium-to-large effect sizes associated with rTMS-induced changes in regional amplitude and graph-theoretic properties, a larger sample would likely detect statistically significant effects after correction for multiple comparisons. Therefore, future research should include larger samples to increase statistical power and improve the validity of the results. In small-sample studies such as the present one, there is also an inherent trade-off between controlling false positives (with more stringent thresholds) and false negatives (with more lenient thresholds) (Jia et al., 2021). To reduce the risk of false negatives in the baseline comparisons between patients and healthy controls, we used a relatively lenient correction threshold. Future research with larger samples may consider using more stringent correction methods to more rigorously control false-positive rates. Second, due to scanner and patient availability, there was variability in the delay intervals between the first MRI scan/clinical evaluation and the start of rTMS, as well as between the end of rTMS and the second MRI scan/clinical evaluation. The post-treatment interval varied considerably among patients (1–20 days), and time interval data were unavailable for the sham group. Although linear regression analysis indicated that variation in post-treatment delay may not be linearly related to clinical ratings or brain activity, future studies should aim to conduct MRI scans and clinical assessments immediately before and after both active and sham interventions, or at least minimize delay variability and record interval data for both groups. This would enable more accurate comparisons of brain and swallowing function by controlling for the potential influence of spontaneous recovery and would help disentangle immediate from longer-term effects. Third, we did not follow up with patients to assess long-term treatment effect (e.g., ≥1 month) to determine whether swallowing improvements were sustained. Fourth, patients in this study had stroke lesions at various locations (cortical, subcortical, or brainstem). Future studies should, if possible, recruit patients with lesions in the same location and hemisphere to avoid the potential confounding effects of lesion variability. Lastly, the videofluoroscopic swallowing study (VFSS) and fiberoptic endoscopic evaluation of swallowing (FEES) are considered gold-standard methods for evaluating dysphagia, as they provide objective and accurate assessments of swallowing function. However, we were unable to perform VFSS on all patients before and after treatment due to practical limitations, and FEES was not available during the study period. Future research should consider incorporating VFSS or FEES, where feasible, alongside clinical scales for a more objective and comprehensive assessment of swallowing function.

Therefore, ideally, future studies should be RCTs that recruit a larger sample—determined through *a priori* power calculation—of patients with the same lesion location. They should also carefully plan the delay intervals between rTMS and MRI/clinical evaluations, and include follow-up assessments to evaluate outcomes over time. Additionally, a more effective sham method is needed to preserve blinding and help reduce attrition, such as the approach used by Mennemeier et al. (2009), in which a sham coil identical in appearance and sound to the active coil was positioned in the same orientation, and electrical stimulation was delivered via scalp electrodes to mimic the cutaneous sensation of TMS. Alternatively, an active control condition could be employed, in which real rTMS is delivered to a brain region considered irrelevant to the disease, making it more difficult for participants to discern whether they are receiving active treatment.

## 6 Conclusion

This exploratory study showed that dysphagic stroke patients treated with 5 Hz rTMS over the contralesional mylohyoid cortical representation experienced greater improvement in swallowing ability, as assessed by clinical scales, compared with patients receiving sham rTMS. We also identified potential neural correlates associated with the effects of our rTMS protocol, which appeared to involve reduced spontaneous activity in the left insula, left middle frontal gyrus (pars orbitalis), right putamen, and right middle frontal gyrus, as well as altered topological properties in the left medial superior frontal gyrus and right middle frontal gyrus. These findings suggest that rTMS may improve dysphagia by modulating the sensorimotor network and inducing neural reorganisation in both the affected and unaffected hemispheres. The results are encouraging but preliminary. The current findings and interpretations require validation in future RCTs with larger samples and longitudinal follow-up.

## Data Availability

The raw data supporting the conclusions of this article will be made available by the authors, without undue reservation.

## Footnotes

1
http://restfmri.net/forum/restplus

2
https://www.fil.ion.ucl.ac.uk/spm/

3
www.graphpad.com
