# Supplementary material for: Effects of rTMS on swallowing function and neuroimaging features in post-stroke dysphagia
Source: Front Hum Neurosci. 2025 Dec 3;19:1573083. doi: 10.3389/fnhum.2025.1573083 (PMC12708527; doi:10.3389/fnhum.2025.1573083)
Supplement: Supplementary file 1 [file Data_Sheet_1.docx]

Supplementary Material

# Supplementary figures

Supplementary Figure 1. σ values (small-world index) plotted across the defined range of sparsity thresholds (0.05 to 0.5) in patients who received rTMS. Each point represents the mean σ value across all patients at that threshold.

Supplementary Figure 2. σ values (small-world index) plotted across the defined range of sparsity thresholds (0.05 to 0.5) in patients who received sham rTMS. Each point represents the mean σ value across all patients at that threshold.


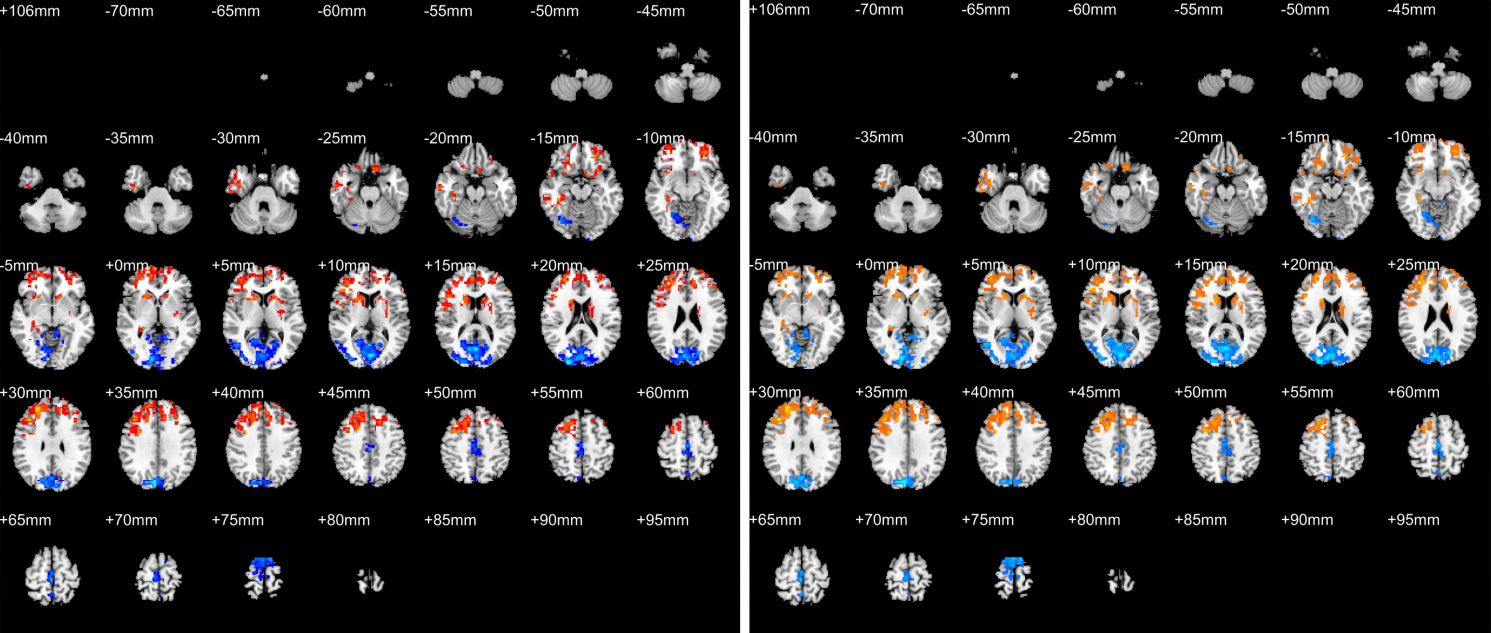
Supplementary Figure 3. Spatial patterns of ALFF differences between baseline (pre-treatment) data of all dysphagic stroke patients and data from healthy controls. Left: results of the original analysis without regressing out mean framewise displacement (FD). Right: results after regressing out mean FD. ALFF = amplitude of low-frequency fluctuation.


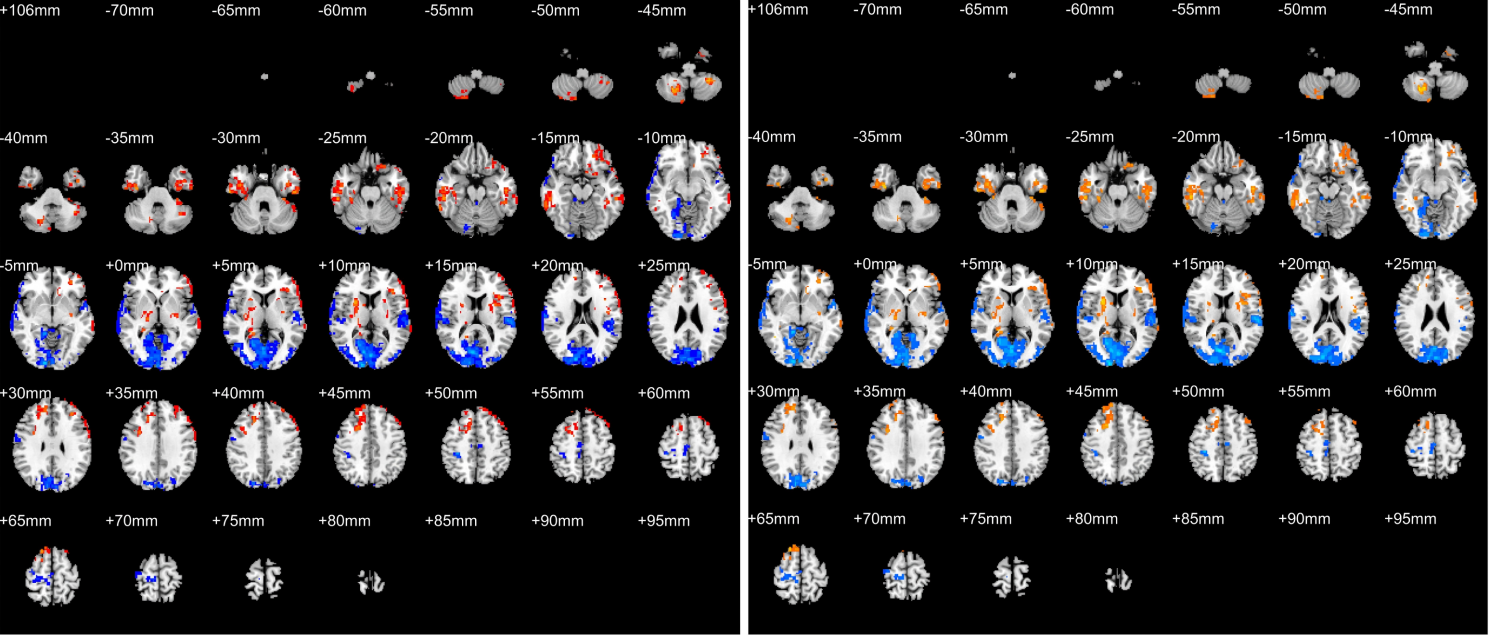
Supplementary Figure 4. Spatial patterns of fALFF differences between baseline (pre-treatment) data of all dysphagic stroke patients and data from healthy controls. Left: results of the original analysis without regressing out mean framewise displacement (FD). Right: results after regressing out mean FD. fALFF = fractional amplitude of low-frequency fluctuation.


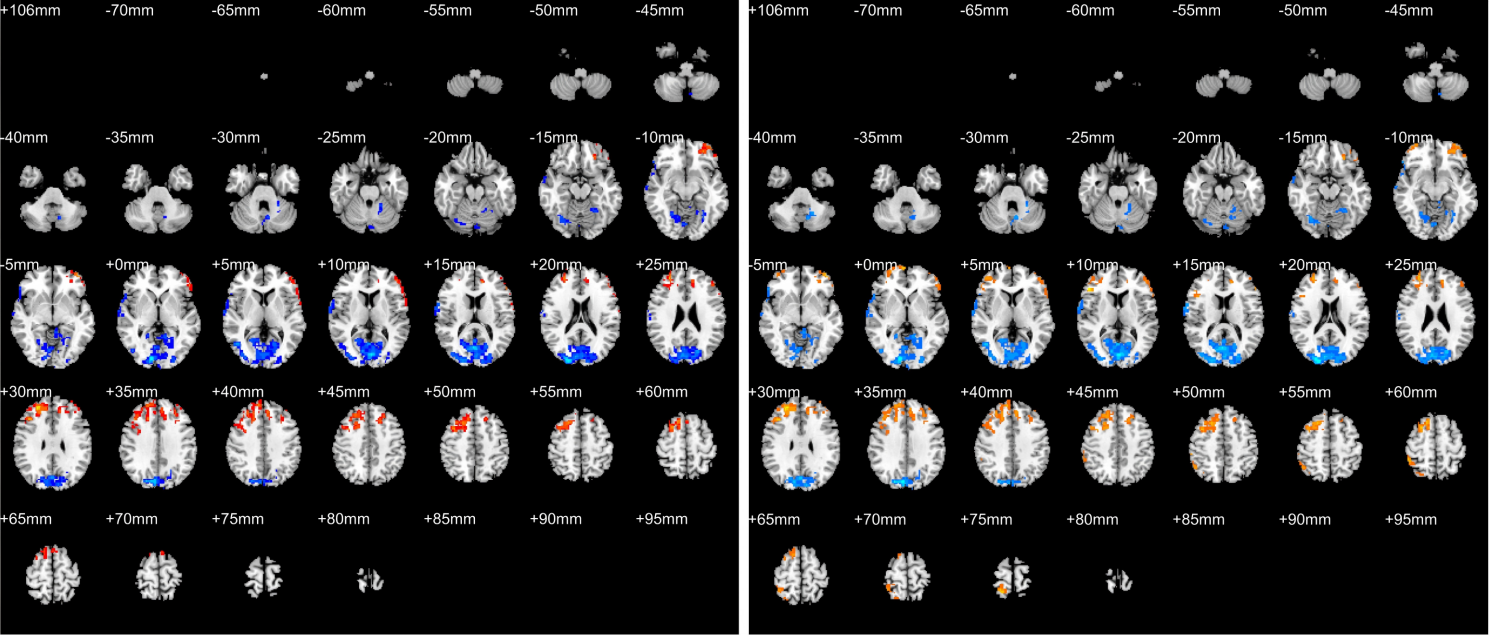
Supplementary Figure 5. Spatial patterns of PerAF differences between baseline (pre-treatment) data of all dysphagic stroke patients and data from healthy controls. Left: results of the original analysis without regressing out mean framewise displacement (FD). Right: results after regressing out mean FD. PerAF = percentage amplitude of fluctuation.

# Supplementary tables

| Supplementary Table 1. Decrease in clustering coefficient of the left medial superior frontal gyrus and right middle frontal gyrus after rTMS treatment (proportional-density window: 0.05–0.4; positive functional connectivity matrix) | | | | | |
| --- | --- | --- | --- | --- | --- |
|  |  |  |  |  |  |
|  |  |  | **Mean (SD)** | |  |
| **Metric** | **Group** | **Brain region** | **Pre-treatment** | **Post-treatment** | **Test statistic** |
| Node - Clustering Coefficient | rTMS | Fusiform_R | 0.04 (0.04) | 0.08 (0.06) | *t* = 1.30, *p* = .22 |
|  |  | Occipital_Sup_R_1 | 0.14 (0.07) | 0.16 (0.06) | *t* = 0.50, *p* = .63 |
|  |  | Putamen_L | 0.08 (0.07) | 0.05 (0.03) | *t* = -1.66, *p* = .13 |
|  |  | Putamen_R | 0.10 (0.08) | 0.05 (0.04) | *t* = -1.63, *p* = .13 |
|  |  | Frontal_Mid_R | 0.11 (0.05) | 0.05 (0.02) | ******t* = -4.03, *p* = .0024 |
|  |  | Supp_Motor_Area_R_1 | 0.06 (0.04) | 0.08 (0.05) | *t* = 1.14, *p* = .28 |
|  |  | Cerebelum_8_R | 0.05 (0.03) | 0.06 (0.06) | *t* = 0.23, *p* = .82 |
|  |  | Temporal_Inf_R | 0.06 (0.04) | 0.08 (0.06) | *t* = 0.56, *p* = .59 |
|  |  | SupraMarginal_L | 0.04 (0.02) | 0.08 (0.06) | *t* = 2.42, *p* = .04 |
|  |  | Cuneus_R | 0.12 (0.05) | 0.12 (0.06) | *t* = -0.43, *p* = .68 |
|  |  | Postcentral_R_1 | 0.05 (0.02) | 0.09 (0.06) | *t* = 2.16, *p* = .06 |
|  |  | Insula_L | 0.07 (0.06) | 0.04 (0.02) | *t* = -1.08, *p* = .31 |
|  |  | Supp_Motor_Area_R_2 | 0.07 (0.05) | 0.07 (0.04) | *t* = -0.28, *p* = .78 |
|  |  | Postcentral_R_2 | 0.07 (0.05) | 0.07 (0.05) | *t* = -0.04, *p* = .97 |
|  |  | Occipital_Sup_R_2 | 0.13 (0.06) | 0.13 (0.05) | *t* = 0.30, *p* = .77 |
|  |  | Rolandic_Oper_R | 0.07 (0.04) | 0.09 (0.06) | *t* = 1.11, *p* = .30 |
|  |  | Frontal_Mid_Orb_L | 0.08 (0.04) | 0.05 (0.06) | *t* = -1.92, *p* = .08 |
|  |  | Frontal_Sup_Medial_L | 0.12 (0.05) | 0.06 (0.03) | ******t* = -4.60, *p* = .001 |
| Node - Clustering Coefficient | Sham rTMS | Fusiform_R | 0.05 (0.05) | 0.06 (0.06) | *t* = 0.49, *p* = .64 |
|  |  | Occipital_Sup_R_1 | 0.13 (0.04) | 0.17 (0.05) | *t* = 1.81, *p* = .10 |
|  |  | Putamen_L | 0.07 (0.05) | 0.07 (0.06) | *t* = -0.29, *p* = .78 |
|  |  | Putamen_R | 0.05 (0.04) | 0.06 (0.07) | *t* = 0.60, *p* = .57 |
|  |  | Frontal_Mid_R | 0.07 (0.03) | 0.09 (0.04) | *t* = 1.44, *p* = .18 |
|  |  | Supp_Motor_Area_R_1 | 0.07 (0.06) | 0.07 (0.04) | *t* = -0.27, *p* = .79 |
|  |  | Cerebelum_8_R | 0.06 (0.06) | 0.03 (0.03) | *t* = -1.59, *p* = .15 |
|  |  | Temporal_Inf_R | 0.08 (0.06) | 0.09 (0.04) | *t* = 0.36, *p* = .73 |
|  |  | SupraMarginal_L | 0.06 (0.05) | 0.05 (0.04) | *t* = -0.53, *p* = .61 |
|  |  | Cuneus_R | 0.11 (0.05) | 0.12 (0.04) | *t* = 0.51, *p* = .62 |
|  |  | Postcentral_R_1 | 0.05 (0.04) | 0.06 (0.05) | *t* = 0.38, *p* = .71 |
|  |  | Insula_L | 0.04 (0.04) | 0.02 (0.03) | *t* = -1.10, *p* = .30 |
|  |  | Supp_Motor_Area_R_2 | 0.08 (0.07) | 0.08 (0.04) | *t* = -0.05, *p* = .96 |
|  |  | Postcentral_R_2 | 0.09 (0.04) | 0.10 (0.04) | *t* = 0.70, *p* = .50 |
|  |  | Occipital_Sup_R_2 | 0.14 (0.06) | 0.16 (0.05) | *t* = 0.80, *p* = .45 |
|  |  | Rolandic_Oper_R | 0.08 (0.04) | 0.07 (0.05) | *t* = -0.31, *p* = .76 |
|  |  | Frontal_Mid_Orb_L | 0.08 (0.08) | 0.08 (0.07) | *t* = -0.09, *p* = .93 |
|  |  | Frontal_Sup_Medial_L | 0.08 (0.06) | 0.08 (0.04) | *t* = -0.01, *p* = .99 |
| Note: Area under the curve was used for statistical analysis of this graph metric. * Not significant after Bonferroni correction for multiple comparisons. Frontal_Mid_R = right middle frontal gyrus; Frontal_Sup_Medial_L = left medial superior frontal gyrus | | | | | |

| Supplementary Table 2. Decrease in local efficiency of the left medial superior frontal gyrus and right middle frontal gyrus after rTMS treatment (proportional-density window: 0.05–0.4; positive functional connectivity matrix) | | | | | |
| --- | --- | --- | --- | --- | --- |
|  |  |  |  |  |  |
| **Metric** | **Group** | **Brain region** | **Pre-treatment** | **Post-treatment** | **Test statistic** |
| Node - Local Efficiency | rTMS | Fusiform_R | 0.05 (0.05) | 0.09 (0.07) | *t* = 1.25, *p* = .24 |
|  |  | Occipital_Sup_R_1 | 0.13 (0.06) | 0.15 (0.05) | *t* = 0.59, *p* = .57 |
|  |  | Putamen_L | 0.09 (0.07) | 0.06 (0.03) | *t* = -1.80, *p* = .10 |
|  |  | Putamen_R | 0.11 (0.08) | 0.06 (0.05) | *t* = -1.39, *p* = .20 |
|  |  | Frontal_Mid_R | 0.12 (0.05) | 0.06 (0.03) | ******t* = -3.95, *p* = .0027 |
|  |  | Supp_Motor_Area_R_1 | 0.06 (0.04) | 0.10 (0.06) | *t* = 1.53, *p* = .16 |
|  |  | Cerebelum_8_R | 0.07 (0.03) | 0.07 (0.07) | *t* = 0.11, *p* = .91 |
|  |  | Temporal_Inf_R | 0.07 (0.04) | 0.09 (0.07) | *t* = 0.53, *p* = .61 |
|  |  | SupraMarginal_L | 0.05 (0.03) | 0.10 (0.07) | *t* = 2.56, *p* = .03 |
|  |  | Cuneus_R | 0.14 (0.05) | 0.13 (0.06) | *t* = -0.74, *p* = .48 |
|  |  | Postcentral_R_1 | 0.07 (0.03) | 0.10 (0.06) | *t* = 1.76, *p* = .11 |
|  |  | Insula_L | 0.08 (0.07) | 0.05 (0.02) | *t* = -1.27, *p* = .23 |
|  |  | Supp_Motor_Area_R_2 | 0.08 (0.05) | 0.08 (0.04) | *t* = 0.37, *p* = .72 |
|  |  | Postcentral_R_2 | 0.09 (0.05) | 0.08 (0.05) | *t* = -0.23, *p* = .82 |
|  |  | Occipital_Sup_R_2 | 0.12 (0.06) | 0.13 (0.05) | *t* = 0.47, *p* = .65 |
|  |  | Rolandic_Oper_R | 0.08 (0.04) | 0.10 (0.05) | *t* = 0.76, *p* = .46 |
|  |  | Frontal_Mid_Orb_L | 0.10 (0.05) | 0.07 (0.08) | *t* = -1.27, *p* = .23 |
|  |  | Frontal_Sup_Medial_L | 0.13 (0.05) | 0.07 (0.04) | ******t* = -4.41, *p* = .0013 |
| Node - Local Efficiency | Sham rTMS | Fusiform_R | 0.06 (0.05) | 0.07 (0.06) | *t* = 0.37, *p* = .72 |
|  |  | Occipital_Sup_R_1 | 0.13 (0.04) | 0.15 (0.05) | *t* = 1.34, *p* = .21 |
|  |  | Putamen_L | 0.08 (0.05) | 0.08 (0.07) | *t* = -0.34, *p* = .74 |
|  |  | Putamen_R | 0.06 (0.05) | 0.07 (0.07) | *t* = 0.41, *p* = .69 |
|  |  | Frontal_Mid_R | 0.08 (0.03) | 0.10 (0.04) | *t* = 1.99, *p* = .08 |
|  |  | Supp_Motor_Area_R_1 | 0.08 (0.06) | 0.08 (0.05) | *t* = -0.07, *p* = .95 |
|  |  | Cerebelum_8_R | 0.08 (0.07) | 0.03 (0.04) | *t* = -1.61, *p* = .14 |
|  |  | Temporal_Inf_R | 0.09 (0.06) | 0.11 (0.04) | *t* = 0.70, *p* = .53 |
|  |  | SupraMarginal_L | 0.06 (0.05) | 0.06 (0.05) | *t* = -0.17, *p* = .87 |
|  |  | Cuneus_R | 0.13 (0.05) | 0.14 (0.06) | *t* = 0.59, *p* = .57 |
|  |  | Postcentral_R_1 | 0.06 (0.04) | 0.07 (0.06) | *t* = 0.44, *p* = .67 |
|  |  | Insula_L | 0.05 (0.05) | 0.02 (0.03) | *t* = -1.31, *p* = .22 |
|  |  | Supp_Motor_Area_R_2 | 0.09 (0.07) | 0.10 (0.04) | *t* = 0.34, *p* = .74 |
|  |  | Postcentral_R_2 | 0.10 (0.04) | 0.13 (0.03) | *t* = 1.55, *p* = .16 |
|  |  | Occipital_Sup_R_2 | 0.13 (0.06) | 0.15 (0.04) | *t* = 0.77, *p* = .46 |
|  |  | Rolandic_Oper_R | 0.08 (0.05) | 0.09 (0.06) | *t* = 0.08, *p* = .94 |
|  |  | Frontal_Mid_Orb_L | 0.08 (0.08) | 0.10 (0.07) | *t* = 0.32, *p* = .75 |
|  |  | Frontal_Sup_Medial_L | 0.09 (0.05) | 0.09 (0.04) | *t* = 0.26, *p* = .80 |
| Note: Area under the curve was used for statistical analysis of this graph metric. * Not significant after Bonferroni correction for multiple comparisons. Frontal_Mid_R = right middle frontal gyrus; Frontal_Sup_Medial_L = left medial superior frontal gyrus | | | | | |

| Supplementary Table 3. Increase in betweenness centrality of the right middle frontal gyrus after rTMS treatment (proportional-density window: 0.05–0.4; positive functional connectivity matrix) | | | | | |
| --- | --- | --- | --- | --- | --- |
|  |  |  |  |  |  |
| **Metric** | **Group** | **Brain region** | **Pre-treatment** | **Post-treatment** | **Test statistic** |
| Node - Betweenness Centrality | rTMS | Fusiform_R | 1.78 (2.24) | 0.85 (1.59) | *t* = -1.02, *p* = .33 |
|  |  | Occipital_Sup_R_1 | 1.43 (2.02) | 1.93 (2.77) | *t* = 0.87, *p* = .41 |
|  |  | Putamen_L | 1.87 (2.37) | 2.24 (2.48) | *t* = 0.31, *p* = .76 |
|  |  | Putamen_R | 1.78 (2.31) | 1.30 (1.53) | *t* = -0.55, *p* = .59 |
|  |  | Frontal_Mid_R | 1.78 (2.03) | 7.05 (3.74) | ******t* = 4.13, *p* = .0021 |
|  |  | Supp_Motor_Area_R_1 | 3.70 (3.66) | 3.23 (3.27) | *t* = -0.47, *p* = .65 |
|  |  | Cerebelum_8_R | 1.29 (1.32) | 0.86 (1.28) | *t* = -0.88, *p* = .40 |
|  |  | Temporal_Inf_R | 3.21 (3.84) | 1.02 (1.37) | *t* = -1.81, *p* = .10 |
|  |  | SupraMarginal_L | 3.92 (4.23) | 1.26 (2.28) | *t* = -2.04, *p* = .07 |
|  |  | Cuneus_R | 2.65 (1.62) | 4.30 (3.84) | *t* = 1.22, *p* = .25 |
|  |  | Postcentral_R_1 | 3.63 (2.47) | 3.21 (2.99) | *t* = -0.38, *p* = .71 |
|  |  | Insula_L | 1.05 (1.05) | 1.48 (2.14) | *t* = 0.79, *p* = .45 |
|  |  | Supp_Motor_Area_R_2 | 3.76 (3.51) | 4.18 (4.42) | *t* = 0.25, *p* = .81 |
|  |  | Postcentral_R_2 | 1.24 (1.85) | 2.31 (2.53) | *t* = 1.14, *p* = .28 |
|  |  | Occipital_Sup_R_2 | 2.13 (2.23) | 3.04 (2.33) | *t* = 0.87, *p* = .40 |
|  |  | Rolandic_Oper_R | 4.74 (3.42) | 1.90 (2.57) | *t* = -2.05, *p* = .07 |
|  |  | Frontal_Mid_Orb_L | 2.66 (3.10) | 3.22 (3.49) | *t* = 0.38, *p* = .71 |
|  |  | Frontal_Sup_Medial_L | 1.93 (1.66) | 4.48 (3.10) | *t* = 2.53, *p* = .03 |
| Node - Betweenness Centrality | Sham rTMS | Fusiform_R | 1.12 (2.55) | 0.69 (1.26) | *t* = -0.44, *p* = .67 |
|  |  | Occipital_Sup_R_1 | 1.99 (2.59) | 1.10 (1.14) | *t* = -1.00, *p* = .34 |
|  |  | Putamen_L | 0.72 (1.23) | 0.71 (1.61) | *t* = -0.01, *p* = .99 |
|  |  | Putamen_R | 1.12 (1.04) | 1.18 (2.17) | *t* = 0.07, *p* = .95 |
|  |  | Frontal_Mid_R | 4.85 (3.99) | 3.79 (4.03) | *t* = -0.60, *p* = .56 |
|  |  | Supp_Motor_Area_R_1 | 5.89 (4.31) | 7.44 (5.36) | *t* = 1.32, *p* = .22 |
|  |  | Cerebelum_8_R | 1.53 (2.12) | 0.66 (0.77) | *t* = -1.11, *p* = .30 |
|  |  | Temporal_Inf_R | 1.69 (2.23) | 2.54 (2.96) | *t* = 0.66, *p* = .53 |
|  |  | SupraMarginal_L | 1.12 (1.67) | 2.10 (2.59) | *t* = 1.10, *p* = .30 |
|  |  | Cuneus_R | 3.49 (3.58) | 3.54 (2.86) | *t* = 0.03, *p* = .98 |
|  |  | Postcentral_R_1 | 2.71 (3.07) | 1.50 (1.37) | *t* = -1.10, *p* = .30 |
|  |  | Insula_L | 0.10 (0.14) | 1.34 (2.18) | *t* = 1.87, *p* = .09 |
|  |  | Supp_Motor_Area_R_2 | 4.91 (4.40) | 4.34 (3.08) | *t* = -0.35, *p* = .73 |
|  |  | Postcentral_R_2 | 1.41 (1.28) | 0.87 (1.61) | *t* = -0.87, *p* = .41 |
|  |  | Occipital_Sup_R_2 | 2.04 (1.46) | 1.76 (1.64) | *t* = -0.40, *p* = .70 |
|  |  | Rolandic_Oper_R | 2.59 (3.37) | 2.18 (2.26) | *t* = -0.27, *p* = .79 |
|  |  | Frontal_Mid_Orb_L | 1.20 (1.38) | 1.64 (2.94) | *t* = 0.41, *p* = .69 |
|  |  | Frontal_Sup_Medial_L | 2.02 (2.38) | 1.99 (2.09) | *t* = -0.02, *p* = .98 |
| Note: Area under the curve was used for statistical analysis of this graph metric. * Not significant after Bonferroni correction for multiple comparisons. Frontal_Mid_R = right middle frontal gyrus | | | | | |

| Supplementary Table 4. Significant results from the pretreatment comparison between patients and healthy controls using GRF (voxel *p* < 0.01, cluster *p* < 0.05) and FDR correction methods. | | | |
| --- | --- | --- | --- |
|  |  |  |  |
| **Metric** | **GRF (voxel *p* < 0.05)** | **GRF (voxel *p* < 0.01)** | **FDR** |
| ALFF | Fusiform_R | Frontal_Mid_R | Calcarine_L |
|  | Occipital_Sup_R | Occipital_Sup_R | Occipital_Sup_R |
|  | Putamen_L | Putamen_R | Frontal_Mid_R |
|  | Putamen_R | Paracentral_Lobule_R |  |
|  | Frontal_Mid_R | Supp_Motor_Area_R |  |
|  | Supp_Motor_Area_R |  |  |
| fALFF | Cerebelum_8_R | Cerebelum_8_R | Cerebelum_8_R |
|  | Temporal_Inf_R | Temporal_Inf_R | Calcarine_R |
|  | SupraMarginal_L | Temporal_Inf_L | Cuneus_R |
|  | Cuneus_R | Cuneus_R | Lingual_R |
|  | Postcentral_R_1 | SupraMarginal_L | SupraMarginal_L |
|  | Insula_L | Frontal_Mid_R |  |
|  | Supp_Motor_Area_R |  |  |
|  | Postcentral_R_2 |  |  |
| PerAF | Frontal_Mid_R | Occipital_Sup_R |  |
|  | Occipital_Sup_R | Frontal_Mid_R |  |
|  | Rolandic_Oper_R | Supp_Motor_Area_R |  |
|  | Frontal_Mid_Orb_L |  |  |
|  | Frontal_Sup_Medial_L |  |  |
| Note: For FDR correction, *q* = 0.05 and cluster size > 10 voxels. GRF = Gaussian random field; FDR = false discovery rate. | | | |
